# Photoreceptor distributions, visual pigments and the opsin repertoire of Atlantic halibut (*Hippoglossus hippoglossus*)

**DOI:** 10.1038/s41598-022-11998-9

**Published:** 2022-05-16

**Authors:** Kennedy Bolstad, Iñigo Novales Flamarique

**Affiliations:** 1 Department of Biological Sciences, Simon Fraser University, Burnaby, BC V5A 1S6 Canada; 2 Department of Biology, University of Victoria, Victoria, BC V8W 2Y2 Canada

**Keywords:** Nervous system, Sensory systems, Visual system, Retina

## Abstract

Fishes often have cone photoreceptors organized in lattice-like mosaic formations. In flatfishes, these lattices undergo dramatic changes during metamorphosis whereby a honeycomb mosaic of single cones in the larva is replaced by a square mosaic of single and double cones in the adult. The spatio-temporal dynamics of this transition are not well understood. Here, we describe the photoreceptors and mosaic formations that occur during the larva to juvenile transition of Atlantic halibut from the beginning of eye migration to its completion. To gauge the possibility of colour vision, visual pigments in juveniles were measured by microspectrophotometry and the opsin repertoire explored using bioinformatics. At the start of eye migration, the larva had a heterogeneous retina with honeycomb mosaic in the dorsonasal and ventrotemporal quadrants and a square mosaic in the ventronasal and dorsotemporal quadrants. By the end of metamorphosis, the square mosaic was present throughout the retina except in a centrodorsotemporal area where single, double and triple cones occurred randomly. Six cone visual pigments were found with maximum absorbance (λ_max_, in nm) in the short [S(431) and S(457)], middle [M(500), M(514) and M(527)], and long [L(550)] wavelengths, and a rod visual pigment with λ_max_ at 491 nm. These pigments only partially matched the opsin repertoire detected by query of the Atlantic halibut genome. We conclude that the Atlantic halibut undergoes a complex re-organization of photoreceptors at metamorphosis resulting in a multi-mosaic retina adapted for a demersal life style.

## Introduction

Flatfishes (order Pleuronectiformes) are peculiar among teleosts in that they undergo a pronounced metamorphosis whereby a bilaterally symmetric, pelagic larva transforms into a flattened, demersal juvenile with both eyes on the same side of the head^1,2^. During this period of transformation, which can last from days to weeks depending on the species^3^, the animal experiences a progressive change in the perception of its surroundings resulting from the shifting visual fields of both eyes and increasing proximity to the ocean floor. In parallel, the diet changes from a preponderance of pelagic plankton consumed by pre-metamorphic larvae to small benthic invertebrates favoured by post-metamorphic juveniles^3^. In some species, like the common sole (*Solea solea*) and the Senegalese sole (*Solea senegalensis*), metamorphosis further entails a shift in circadian rhythm from diurnal to nocturnal^4^.

Despite these profound alterations to the anatomy and ecology of flatfishes, their visual system during metamorphosis has seldom been examined and is poorly understood. Morphological investigations into the larval retina of winter flounder, *Pseudopleronectes americanus*, and Atlantic halibut, *Hippoglossus hippoglossus*, have shown the presence of a single morphological type of cone photoreceptor prior to the start of eye migration^5,6^. This cone forms a honeycomb (or hexagonal) mosaic throughout the retina characterized by six neighbours surrounding each photoreceptor. Microspectrophotometric measurements from cone photoreceptors of pre-metamorphic winter flounder larvae revealed a single visual pigment with maximum wavelength of absorption (λ_max_) at 519 nm^7^. Post-metamorphosis, the retina comprised two types of morphological cone types (single and doubles) forming a square mosaic whose repeating unit consisted of a single, centre cone, surrounded by four double cones (the latter made up of two cones apposed together sharing a double membrane partition)^5^. The visual pigment in the single cones peaked at 457 nm, whereas the double cones could be equal members, with a visual pigment peaking at 531 nm, or unequal, with one member possessing a visual pigment with λ_max_ at 531 nm and the other at 547 nm^7^. Thus, the retina of winter flounder transformed its cone mosaic and visual pigment repertoire during metamorphosis. In addition, rod photoreceptors were only present after metamorphosis, with a visual pigment peaking at 506 nm^7^.

Further work using *in-situ* hybridization with riboprobes against the various opsin (visual pigment protein) mRNAs in winter flounder showed a single *rh2* opsin being expressed in the cones prior to metamorphosis and four others (*sws2*, *rh2*, *lws* and *rh1*) post-metamorphosis^8^. In the post-metamorphic flounder, single cones expressed *sws2* transcript whereas double cones expressed *rh2* or *lws* (a single opsin transcript per member) and rods expressed *rh1*. By raising larvae in water containing 3H thymidine and analyzing its retinal uptake, these authors further concluded that double cones central to the peripheral growth zone should form from coalescing single cones^8^. As such, two mechanisms of square mosaic formation would exist during metamorphosis of flatfishes: one operating at the peripheral growth zone producing a square mosaic from differentiation of progenitor cells, and the other more centrally, requiring the re-arrangement of pre-existing, differentiated single cones.

In contrast to the findings in winter flounder, *in-situ* hybridization observations from Atlantic halibut larvae toward the end of yolk sac absorption (i.e., 40 days post-hatching, or about 30 days prior to the start of eye migration) showed expression of four opsin transcripts (*sws1*, *sws2*, *rh2* and *lws*) in the single cone retina^9^. The vast majority (> 90%) of cones expressed *rh2* whereas *sws2* and *lws* were only found in about 10% of photoreceptors. Nonetheless, like *rh2*, *sws2* and *lws* were expressed throughout the retina, whereas *sws1* was restricted to the ventral retina^9,10^. A square mosaic formation showing *rh2* and *lws* transcript expression was later reported in the juvenile (6 month old) retina^11^. Thus, the chromatic organization of photoreceptors during the time of metamorphosis in this species remains unknown. Furthermore, although the appearance of double cones and a square mosaic were reported by morphology around the start of eye migration, and such mosaic was present in the central retina 30 days later (100 days post-hatching)^6^, the spatial dynamics of square mosaic formation remain unresolved.

This study was undertaken to reveal the morphological organization of photoreceptors during the larval to juvenile transition of Atlantic halibut, from the start of eye migration to its completion. Combining these observations and morphometric measures of the lens, the spatial resolving power was derived for different regions of the retina. Microspectrophotometric measurements of visual pigment absorbance from post-metamorphic fish and opsin bioinformatic analyses were also undertaken to gain insight into photoreceptor chromatic diversity and opsin evolution with respect to other flatfishes.

## Results

### Eye morphology and migration

**Figure 1:**
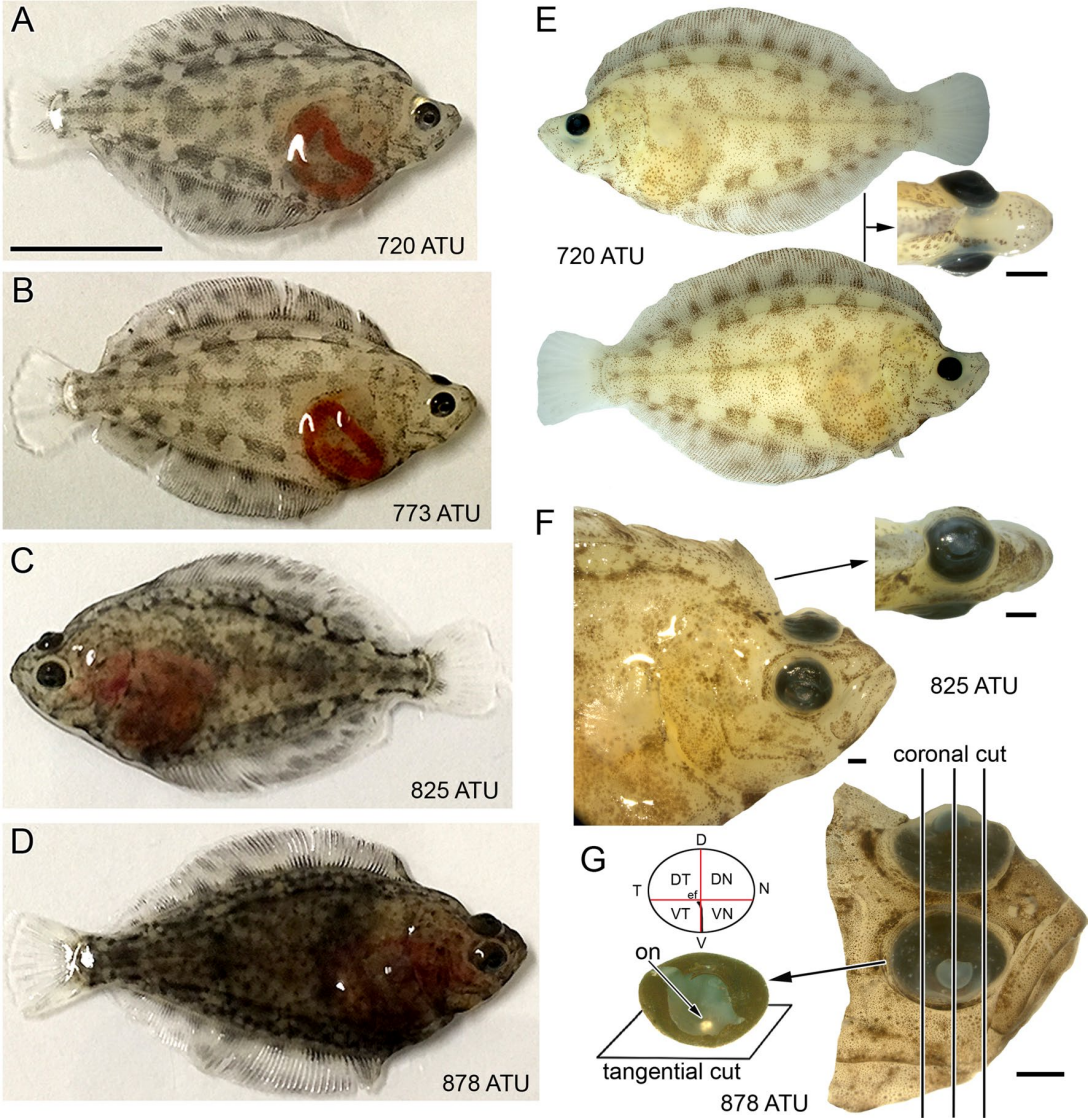
Developmental stages of Atlantic halibut examined illustrating eye migration and orientation of histological sectioning. (**A**–**D**) Body morphology of live fish at approximately 720 ATU (82 days post-hatching) (**A**), 773 ATU (89 days post-hatching) (**B**), 825 ATU (96 days post-hatching) (**C**), and 878 ATU (103 days post-hatching) (**D**). The left eye is migrating in all fish except for the one shown in (**C**), where the right eye is migrating. (**E**) Lateral and top views of a fixed specimen at 720 ATU. Migration of the left eye has just started. (**F**) Lateral and top views of a fixed specimen at 825 ATU. The left eye has migrated half way between the blind side (bottom part of the fish following metamorphosis) and ocular side (top part of the fish following metamorphosis). (**G**) Head of fixed specimen at 878 ATU showing completed eye migration and the extracted right eye without lens. The optic nerve (on) exits the retina below the crossing of theoretical short and long ellipse axes that characterize this elliptically-shaped eye and retina. The optic nerve head coincides with the central end of the embryonic fissure (ef) which extends to the ventral periphery. The panel illustrates the direction of sectioning when full eyes were cut, revealing tangential sections of central retina and more oblique sections toward the periphery, and coronal cuts when a head was sectioned, revealing radial views of the retina throughout the lens region and tangential views at the nasal and temporal ends. The schematic shows the division of a right eye retina into quarters (VT, ventrotemporal; VN, ventronasal; DT, dorsotemporal; DN, dorsonasal). Other abbreviations: V, ventral; D, dorsal; N, nasal; T temporal. Scale bar in (A) equals 1 cm and holds for (**B**–**D**) and the lateral views in (**E**); the other scale bars represent 1 mm and are associated with the figures closest to them.

The youngest Atlantic halibut examined (96 days post-fertilization or 720 ATU; ATU are the Accumulated Temperature Units, i.e. the product of mean rearing water temperature (7.5 ˚C) and days from fertilization, 96) were bilaterally quasi-symmetrical with a compressed, oval shaped body and similar pigmentation on either side of the body (Fig. 1A,E). In the majority of animals examined (6 of 10), one of the eyes (usually the left eye) had recently started migrating as evidenced by its slightly closer proximity to the midline with respect to the other eye (Fig. 1E). Migration of the eye proceeded throughout the study period (Fig. 1A–D) such that between 773 and 825 ATU (i.e., 103 and 110 days post-fertilization, respectively) the migrating eye had reached the mid-line (Fig. 1C,F) or proceeded beyond, to the other side of the head. The oldest halibut examined (878 ATU, or 117 days post-fertilization) had both eyes on the same side of the head (the ocular side, Fig. 1D,G) whereas the opposite side (the blind side) rested on the bottom and had reduced pigmentation.

All fish examined had elliptically shaped eyes and retinas (Fig. 1). The eye dimensions increased from a mean ± SD (n = 10) of 1.6 ± 0.18 mm for the long axis and 1.3 ± 0.11 mm for the short axis at 960 ATU to 2.2 ± 0.13 mm and 1.6 ± 0.15 mm, respectively, at 1170 ATU. The mean lens diameter ± SD (n = 10) increased from 0.44 ± 0.04 mm to 0.64 ± 0.02 mm during the same time period. The extracted retinas showed an embryonic fissure that projected from the medial ventral periphery to the central retina (location of the optic nerve head) pointing slightly to the temporal retina. The optic nerve head was localized in the ventral half of the retina (Fig. 1G), below the theoretical crossing of short and long retinal axes.

### Photoreceptor types and mosaics

**Figure 2:**
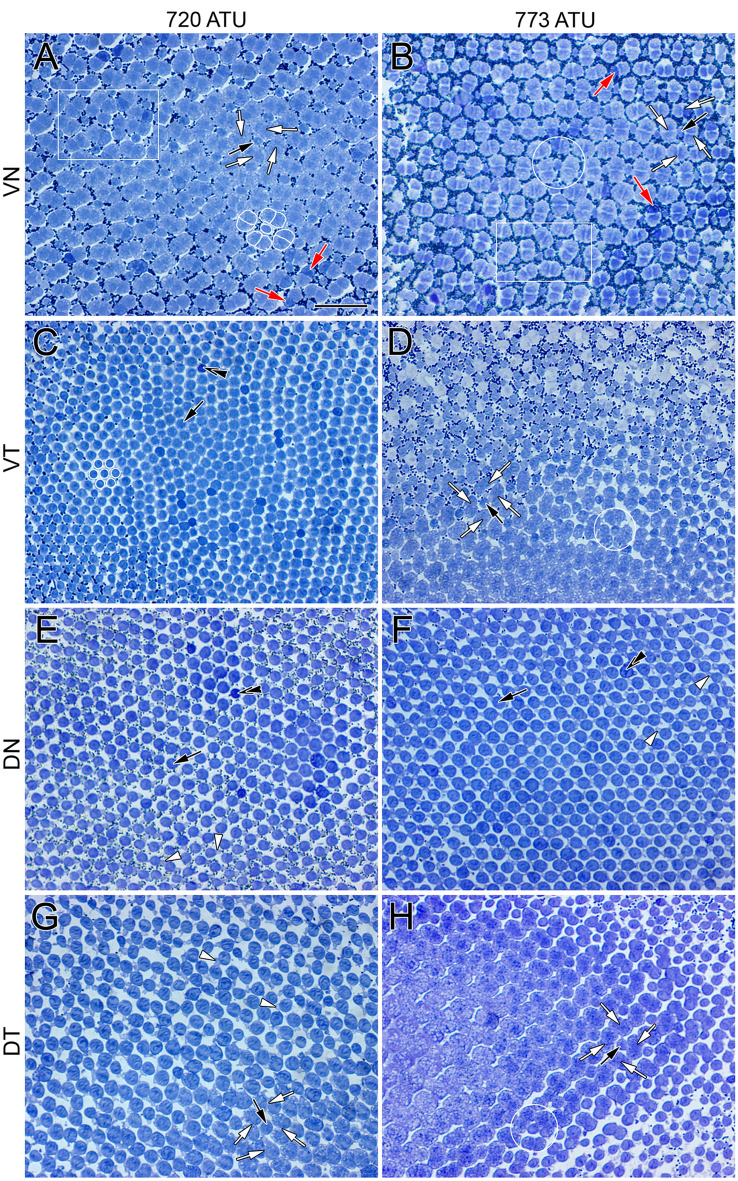
Micrographs of tangential EPON sections showing cone distributions at the level of largest ellipsoid cross section or near the base of the outer segments from various regions of the light adapted retina of Atlantic halibut undergoing metamorphosis. (**A**,**C**,**E**,**G**) Central area from the ventronasal (**A**), ventrotemporal (**C**), dorsonasal (**E**) and dorsotemporal (**G**) retina at 720 ATU. (**B**,**D**,**F**,**H**) Equivalent central sectors to those in (**A**,**C**,**E**,**G**) but from a retina at 773 ATU. At 720 ATU, the ventronasal (**A**) and dorsotemporal (**D**) quadrants show cone square mosaics whereas honeycomb mosaics characterize the ventrotemporal (**C**) and dorsonasal (**E**) quadrants. (**A**) A square mosaic unit consisting of a single cone surrounded by four double cones is traced in white; white arrows point to the apposing partitions of double cone members surrounding a single cone (black arrow); red arrows indicate double cone members that stain darker than their counterparts. The area within the white rectangle illustrates a less regular arrangement of single and double cones. (**C**) A honeycomb mosaic unit consisting of one single cone surrounded by six others is shown in white; double black arrowheads point to a single cone that stains darker than most others in the field of view. (**E**,**G**) White arrowheads point to accessory outer segments of individual single cones. At 773 ATU (**B**,**D**,**F**,**H**), only the central region of the dorsonasal quadrant retains a honeycomb mosaic (**F**). The white rectangle in (**B**) illustrates an area with less regular disposition of single and double cones. (**B**,**D**,**H**) White circles encompass a single cone flanked by a triangular arrangement of three neighbouring double cones. Abbreviations as per Fig. 1. Scale bar in (**A**) equals 10 µm and holds for all panels.

The morphological trends were the same for the migrating and non-migrating eye. At 720 ATU, the retina had two main types of cone photoreceptors: single and double cones (Fig. 2A,C,E,G). The single cones were round in cross section and formed a honeycomb mosaic in the ventrotemporal and dorsonasal regions of the retina (Fig. 2C,E). The honeycomb mosaic consisted of six neighbouring cones flanking a single cone (Fig. 2C; white contours) thereby forming a hexagon if the centres of neighbouring cones were to be connected. The inner segments of some single cones stained darker (Fig. 2C,E; double black arrowheads) than the rest of the cone population, and an accessory outer segment was prominent at the base of the outer segments (Fig. 2E; white arrowheads).

In the ventronasal and dorsotemporal quadrants, double cones were present as part of square mosaics of varying regularity (Fig. 2A,G). The basic square mosaic unit consisted of four double cones surrounding a single (centre) cone (Fig. 2A; white contours). There was, however, substantial deviation from a lattice formation (Fig. 2A; white rectangle). Single (corner) cones, i.e., those facing the partitioning membranes of neighbouring double cones, were absent or extremely rare. Some double cone members in the ventronasal retina stained darker than their counterparts (Fig. 2A; red arrow) and, in the dorsotemporal retina, accessory outer segments were prominent and appeared to make contact between cone types (Fig. 2G; white arrowheads).

At 773 ATU, double cones were present in the centroventrotemporal retina forming a square mosaic region of varying disposition (Fig. 2D). The honeycomb mosaic was restricted to the dorsonasal retina (Fig. 2F). In regions with double cones, an arrangement consisting of a single cone flanked by three double cones was common (Fig. 2B,D,H; white circles), as were irregular formations of single and double cones (Fig. 2B, white rectangle).

**Figure 3:**
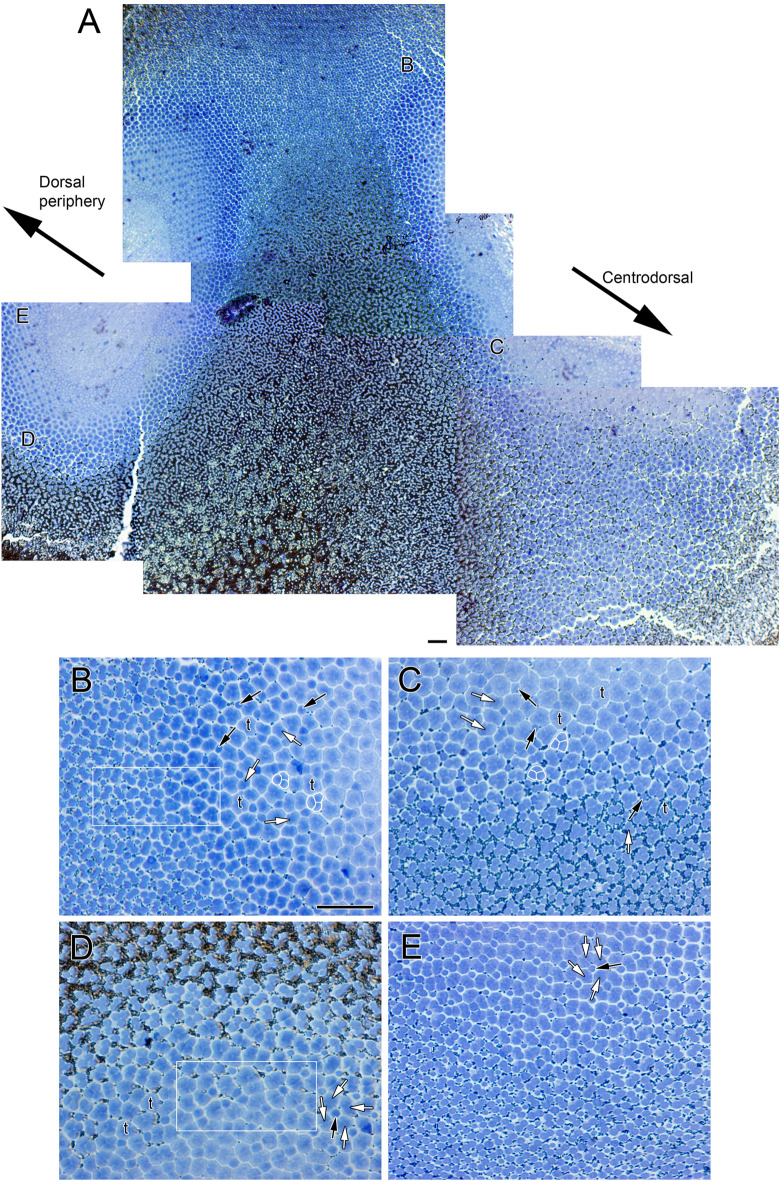
Micrographs of tangential EPON sections showing cone distributions from the dorsotemporal retinal quadrant at 878 ATU. (**A**) View stitched together from multiple micrographs spanning the central to peripheral retina. The centrotemporal retina comprises three different cone types (single, double and triple cones) arranged in various formations whereas the retinal periphery shows a square mosaic. (**B**–**E**) Cone distributions from different areas of the field shown in (**A**) and indicated by the same letters (in **A**). (**B**,**D**) Cone distributions at interfaces between the more central, triple cone-occupied area and the peripheral, square mosaic area (white rectangles); triple cones (t, white contours) are common among double and single cones toward the central retina. (**C**) Area of central retina illustrating larger cells and prominent triple cone presence. (**E**) Square mosaic toward the periphery of the retina. Other symbols and abbreviations as per Fig. 2. Scale bars in (**A**) and (**B**) equal 10 µm; scale bar in (**B**) holds for (**C**–**E**).

**Figure 4:**
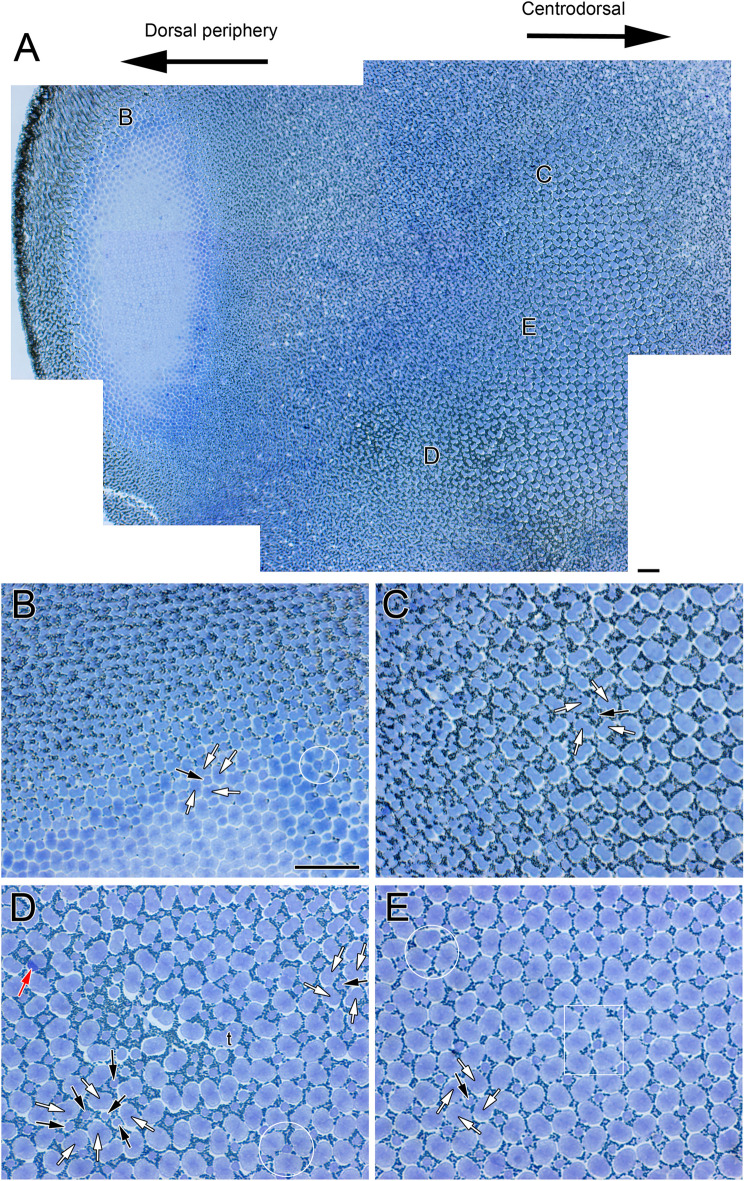
Micrographs of tangential EPON sections showing cone distributions from the dorsonasal retinal quadrant at 878 ATU. (**A**) View stitched together from multiple micrographs spanning the central to peripheral retina. The primary cone formation is a square mosaic but there are other cone arrangements. (**B**–**E**) Cone distributions from different areas of the field shown in (**A**) and indicated by the same letters (in **A**). (**B**,**D**) White circles encompass a single cone flanked by three double cones. (**E**) The white rectangle shows an area where five double cones surround a single cone. Other symbols and abbreviations as per Fig. 2. Scale bars in (**A**) and (**B**) equal 10 µm; scale bar in (**B**) holds for (**C**-**E**).

**Figure 5:**
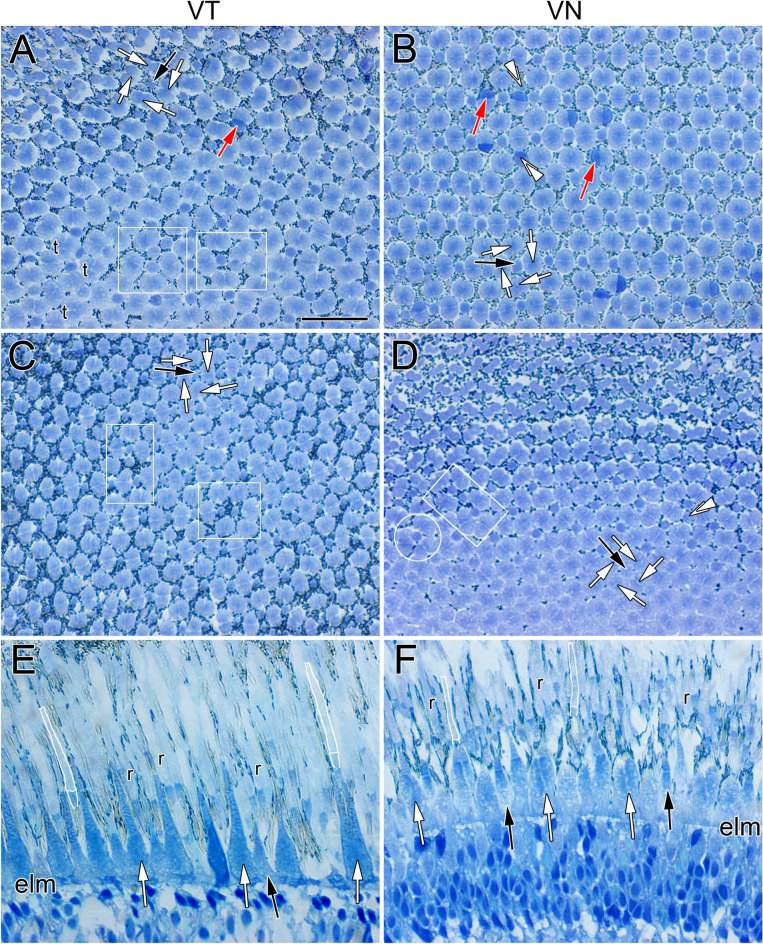
Micrographs of EPON sections showing cone distributions from the ventral retina at 878 ATU. (**A**–**D**) Tangential sections of central retina from the ventrotemporal (**A**) and ventronasal (**B**) quadrants and corresponding sections from the ventrotemporal (**C**) and ventronasal (**D**) periphery. White rectangles in (**A**,**C**,**D**) show cone arrangements that deviate from the square mosaic and the white circle in (**D**) illustrates a single cone surrounded by three double cones. The double white arrowheads in (**B**,**D**) point to single corner cones, which face the partitions of adjacent double cones. (**E**,**F**) Radial sections from the central regions of the ventrotemporal (**E**) and ventronasal (**F**) quadrants; white contours depict rods Abbreviations: r, rod; elm, external limiting membrane. Other symbols and abbreviations as per Fig. 2. Scale bar in (**A**) equals 10 µm and holds for (**B**–**F**).

By the time eye migration was complete (878 ATU), the honeycomb mosaic was absent from the retina and other cone formations had emerged (Figs. 3, 4 and 5). A new cone type, the triple cone (t) (white contours), spanned the centrodorsotemporal retina (Fig. 3A–D) abutting onto a lattice square mosaic of smaller cones closer to the periphery (Fig. 3A,B,E; white rectangle in B). The triple cones were part of mosaic formations that also included single and double cones (Fig. 3B–D) with ratios of double to single cone and double to triple cone that varied between 1.3 and 0.96, respectively, for the centrodorsotemporal retina, to 2.0 and 62 for the dorsonasal retina. The dorsonasal retina consisted primarily of square mosaic formations with larger cones in the central retina and smaller ones toward the periphery (Fig. 4). Although variation in regularity of the square lattice occurred in various areas (Fig. 4B,D,E; white rectangle and circles), cones in the periphery were more tightly packed and the mosaic appeared more regular (Fig. 4B). Cone distributions differed the most from a lattice formation in the region approaching the ventronasal retina, where double cone members with darker stain (red arrow) were present (Fig. 4D).

As per the trends in the dorsonasal retina, the ventral retina at 878 ATU consisted of square mosaics with variable jitter in the lattice (Fig. 5A,C,D; white rectangles and circle). Double cone members with darker stain (red arrows) than their counterparts were more frequent in the ventral retina than in other areas of the retina, as were single corner cones (double white arrowheads) (Fig. 5A,B,D). At this developmental stage, rod photoreceptors were present; their outer segments were larger and displaced further into the retinal pigment epithelium with respect to those of cones (white closed contours, Fig. 5E,F).

**Figure 6:**
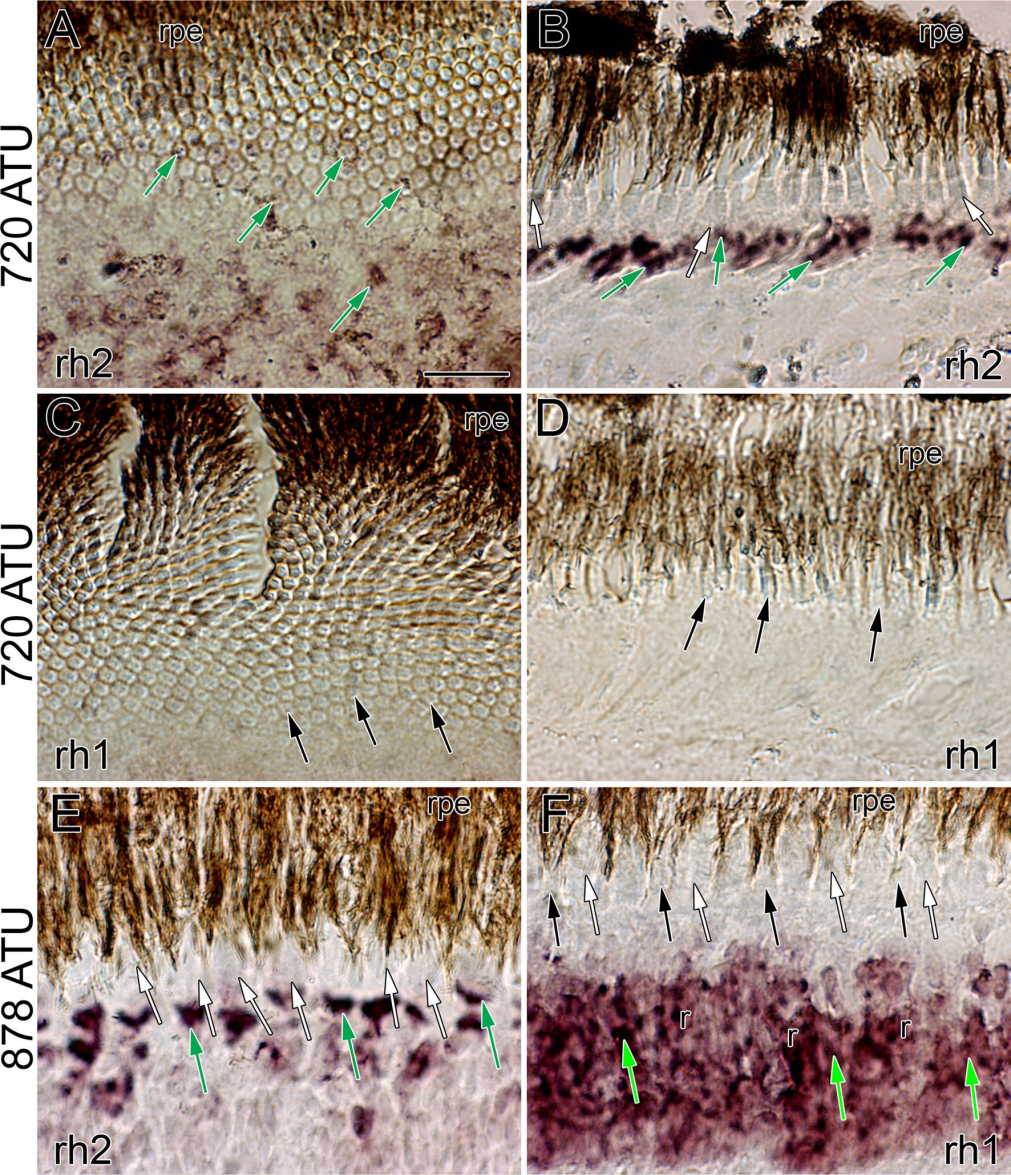
Micrographs of cryosections subjected to *in-situ* hybridization with the *rh1* and *rh2* riboprobes. (**A**) Tangential micrograph from the dorsonasal retina at 720 ATU showing labeling of *rh2* transcripts among single cones of the honeycomb mosaic (green arrows). (**B**) Radial micrograph from a square mosaic region of the centroventral retina showing prominent labeling of *rh2* transcripts (green arrows). (**C**,**D**) Tangential (**C**) and radial (**D**) micrographs from the dorsonasal retina at 720 ATU illustrating lack of *rh1* label among cones of the honeycomb mosaic. (**E**,**F**) Radial micrographs of the retina at 878 ATUs showing *rh2* label of double cones (green arrows) (**E**) and *rh1* label of rods (r) (**F**). The *rh1* label is displaced vitreally (light green arrows) with respect to that of *rh2* in accordance with the position of rod myoids relative to those of cones in the light adapted retina. Other abbreviations and symbols as per Fig. 5. Scale bar in (**A**) equals 10 µm and holds for (**B**–**F**).

To confirm that rod photoreceptors were associated with the appearance of double cones and square mosaic formation, *in-situ* hybridization experiments were performed with two previously established riboprobes, one tagging *rh2* opsin transcript and the other rod (*rh1*) opsin transcript^9^. At all locations, whether characterized by the honeycomb mosaic or by the presence of double cones, the retina of Atlantic halibut at 720 ATU labelled prominently with the *rh2* riboprobe along the inner segment myoids of cones (Fig. 6A,B; green arrows). In contrast, the *rh1* riboprobe failed to label cryosections characterized by a honeycomb mosaic (Fig. 6C,D). At 878 ATU, both the *rh2* and *rh1* riboprobes labelled, respectively, the myoid region of double cone members (Fig. 6E) and those of rods (Fig. 6F). Parallel experiments with corresponding sense probes resulted in no labelling (Supplementary Fig. S1).

### Cone densities and measures of visual acuity

**Figure 7:**
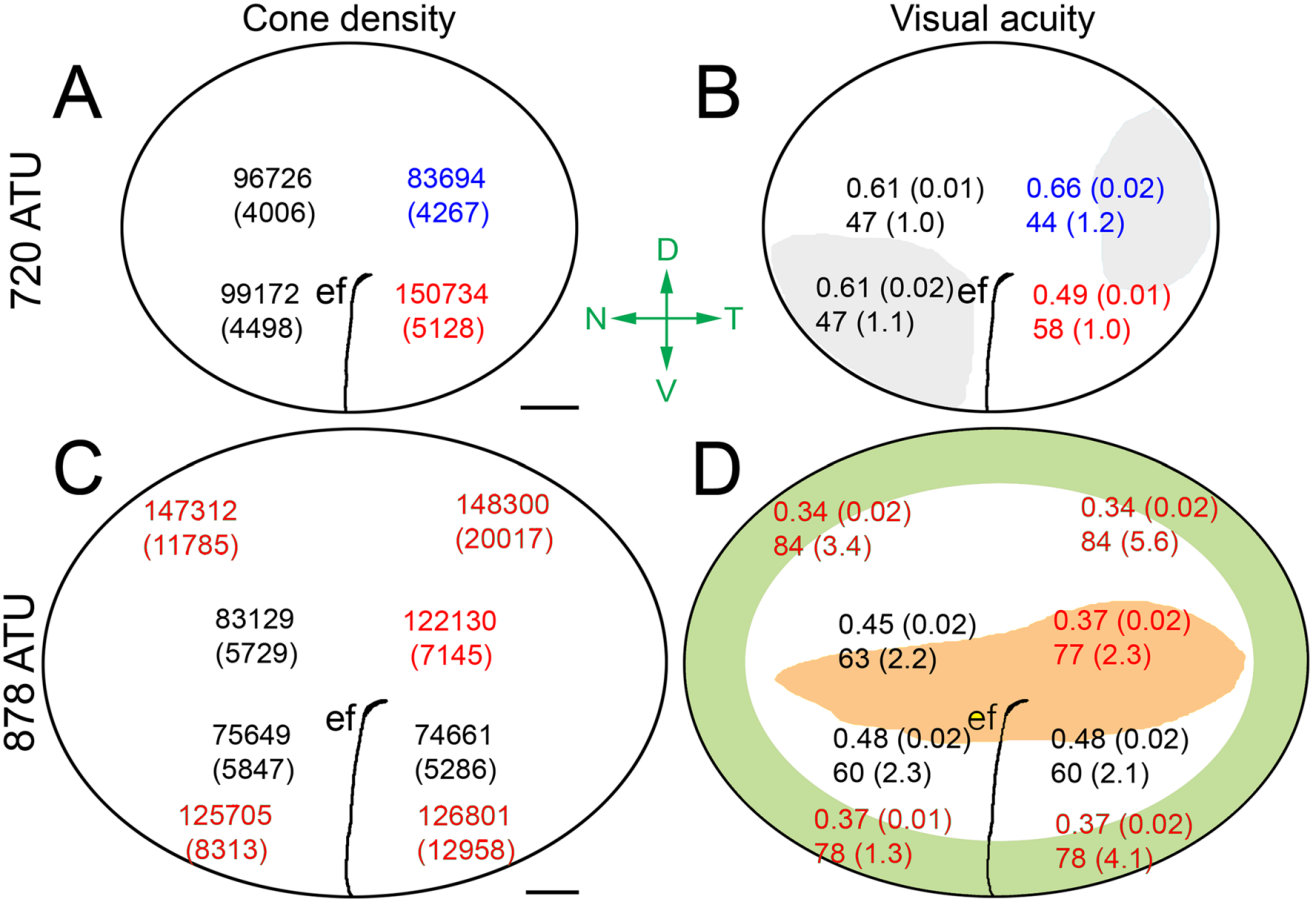
Diagrams illustrating topographic maps of cone densities and visual acuity-related variables extracted from the retinas of four Atlantic halibut at 720 ATU or 878 ATU. Each statistic is the mean (± SD). (**A**,**C**) Cone density per mm^2^; gray zones in (**C**) delineate the approximate areas of square mosaic presence. (**B**,**D**) Minimum resolvable angle (º) (top number), and distance (in mm) (bottom number) at which a 0.5 mm target would be resolved. The green and pink zones in (**D**) denote the approximate areas associated with the peripheral square mosaic and triple cone presence, respectively. Means with different colour are statistically different at α = 0.05 level of significance. The embryonic fissure (ef) runs from the ventral retina toward the centro-temporal retina. Abbreviations (axes in green): D, dorsal; V, ventral; N, nasal; and T, temporal. Scale bars in (**A**) and (**C**) equal 0.2 mm.

A summary topographical schematic showing mean cone densities in the Atlantic halibut retina at the two extreme developmental time periods examined (720 and 878 ATU) revealed profound retinal transformations (Fig. 7). At 720 ATU, the greatest cone density within the central retina was found in the ventrotemporal quadrant and the lowest in the dorsotemporal quadrant (Fig. 7A). Correspondingly, the greatest theoretical spatial resolving power was found in the ventrotemporal quadrant and the lowest in the dorsotemporal quadrant (Fig. 7B). This translated into a mean increase of 14 mm in the distance at which the ventrotemporal retina would resolve a 0.5 mm target with respect to the dorsotemporal retina (Fig. 7B). Based on total density of cones (where each cell was counted individually), the greatest resolving power was found in an area devoid of double cones and square mosaics.

At 878 ATU, the region of the central retina with the greatest cone density had shifted to the dorsotemporal quadrant (Fig. 7C). This region, with theoretical resolving power similar to that of the peripheral retina, also had the greatest density of triple cones (Fig. 7D). Despite an overall reduction in cone density in the central retina during development from 720 to 878 ATU (Fig. 7A,C), theoretical resolving power generally improved (Fig. 7B,D) as a result of increased lens size.

### Visual pigments

**Figure 8:**
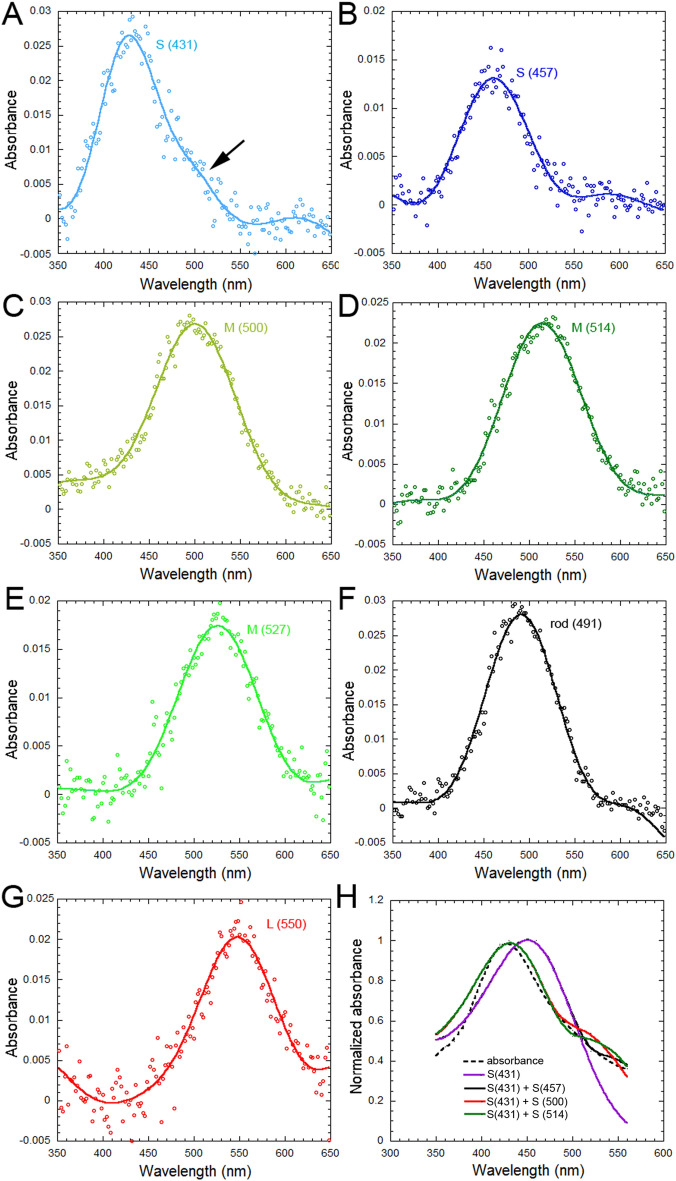
Representative visual pigment absorbance spectra from isolated photoreceptors in the retina of Atlantic halibut juvenile (each trace is the mean of 4–14 records from 4 fish). (**A**) Coexpression of a short wavelength sensitive [S (431)] visual pigment with another visual pigment (as revealed by the “hump” in absorbance at longer wavelengths, black arrow). (**B**) Short wavelength sensitive [S(457)] visual pigment. (**C**) Middle wavelength [M (500)] visual pigment. (**D**) Middle wavelength [M (514)] visual pigment. (**E**) Middle wavelength [M (527)] visual pigment. (**F**) Rod (491) visual pigment. (**G**) Long wavelength sensitive [L(550)] visual pigment. The wavelength of maximum absorbance, λ_max_, associated with each visual pigment type is in parenthesis. (**H**) Simplex-derived fits to the S(431) absorbance in (**A**) (dotted black function) using one [S(431)] or two visual pigment templates (see Table 1 for corresponding fit parameters).

Absorbance measurements from the outer segment of individual photoreceptors from four juvenile Atlantic halibut revealed six cone visual pigments and one rod visual pigment (Fig. 8). The cone visual pigments consisted of two short wavelength (S) types with maximum wavelength of absorbance (λ_max_) ± SD at 431 ± 7 nm (n = 8) (Fig. 8A) and 457 ± 6 nm (n = 7) (Fig. 8B), three middle wavelength (M) types with λ_max_ ± SD at 500 ± 5 nm (n = 23) (Fig. 8C), 514 ± 8 (n = 35) (Fig. 8D), and 527 ± 6 (n = 19) (Fig. 8E), and one long wavelength (L) type peaking at 550 ± 11 nm (n = 4) (Fig. 8G). The rod visual pigment had λ_max_ ± SD at 491 ± 7 nm (n = 25) (Fig. 8F). The S visual pigments were located in the single cones whereas the M and L visual pigments were mostly confined to the double cones, one per double cone member. The S(431) absorbance curve showed an increase (“hump”) between 450 and 550 nm (Fig. 8A). Fitting of this curve with combinations of visual pigment templates (Fig. 8G) suggested that it consisted of the S(431) visual pigment and a smaller amount of either M(500) or M (514) visual pigment (Table 1). The majority of double cones measured were M/M pairs, either 500/514 (n = 18), 514/527 (n = 12), or 527/527 (n = 10), with a minority of M/L pairs (527/550, n = 4). The L/M pairs tended to be shorter size cones, which were more prevalent in the ventral retina.

**Table 1:** Best-fit Simplex derived parameters and corresponding least sum of squares (SS) for the spectral functions depicted in Fig. 8H. The k_431_ and k_other_ coupling constants are the inputs of the visual pigments to the absorbance function [k_431_ corresponds to the S(431) visual pigment and k_other_ to the other pigment in a combination]. The λ_max_ of the visual pigments used in the fitting process are indicated in the first column. The SS statistic is derived from the sum of differences between mean absorbance data and the corresponding values predicted by the Simplex algorithm.

Visual pigments	K_431_	K_other_	*p*	SS
S (431)	1.124	0	1.025	0.333
S (431), S(457)	1.925	0.007	6.494	0.176
S (431), M (500)	1.398	0.061	5.523	0.056
S (431), M(514)	1.263	0.091	3.911	0.059
S (431), M (527)	0.992	0.355	1.689	0.173

### Spatial analyses of cone distributions

**Figure 9:**
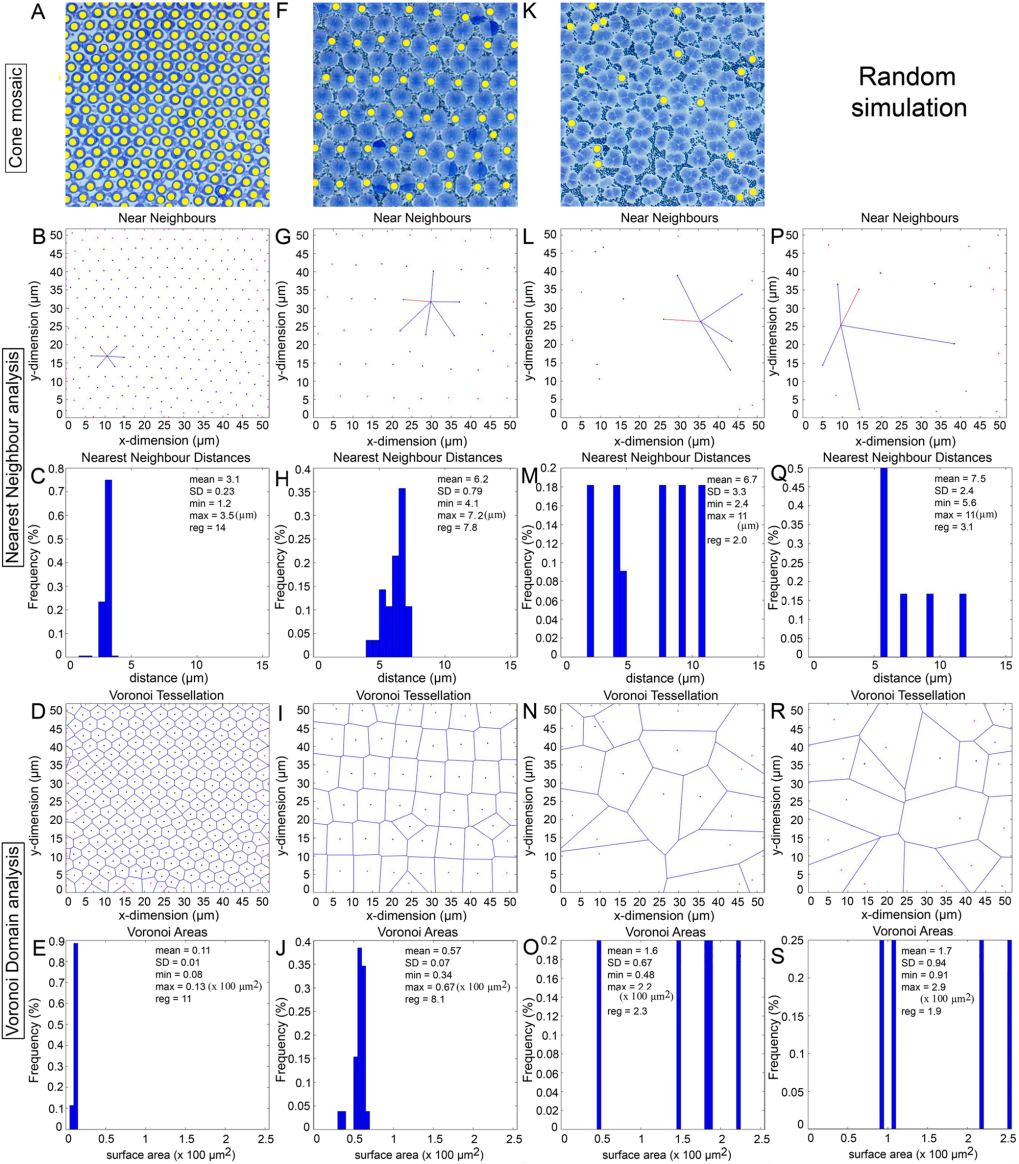
Spatial analysis of single cone distributions from representative mosaics in the retina of Atlantic halibut undergoing metamorphosis. Single cones are marked with yellow dots. (**A**–**E**) Cone mosaic from the centrodorsonasal retina at 773 ATU (**A**); Nearest neighbour analysis of double cone centroids, illustrating the near neighbours of a single cone, including its nearest neighbour in red (**B**) and their frequency distribution (**C**) [statistics (in µm) are the mean nearest neighbour distance, its standard deviation (SD), the minimum (min) and maximum (max) nearest neighbour distances, and the regularity index = mean/SD]; Voronoi tessellation of double cone domains (**D**) and their frequency distribution (**E**) [statistics (in 100 µm^2^) are the mean area (domain), its standard deviation (SD), the minimum (min) and maximum (max) areas, and the regularity index = mean/SD]. (**F**–**J**) Same presentation of data as per (**A**–**E**) but for the centroventronasal retina at 878 ATU. (**K**–**O**) Same presentation of data as per (**A**–**E**) but for the centrodorsotemporal retina at 878 ATU. (**P**–**S**) Same presentation of data as per (**B**–**E**) but for a random distribution of points matched for density with that in (**K**) and constrained by single cone soma size.

**Figure 10:**
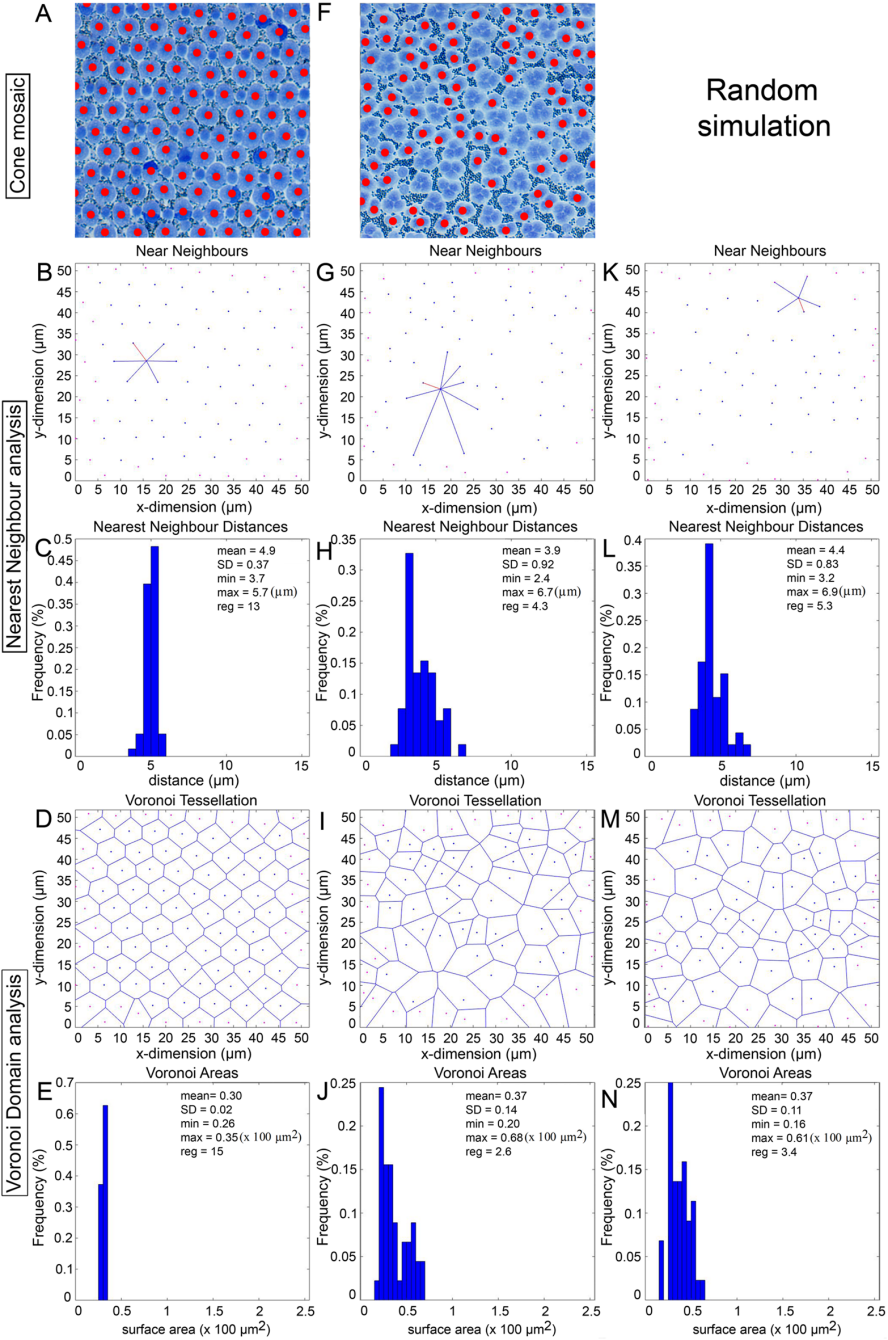
Spatial analysis of double cone distributions from the square mosaic (**A**) and mosaic containing triple cones (**B**), corresponding to (**F**) and (**K**) of Fig. 9. Double cones are marked with red dots. (**K**–**N**) Same presentation of data as per (**G**–**J**) but for a random distribution of points matched for density with that in (**G**) and constrained by double cone soma size. Presentation of data as in Fig. 9.

**Figure 11:**
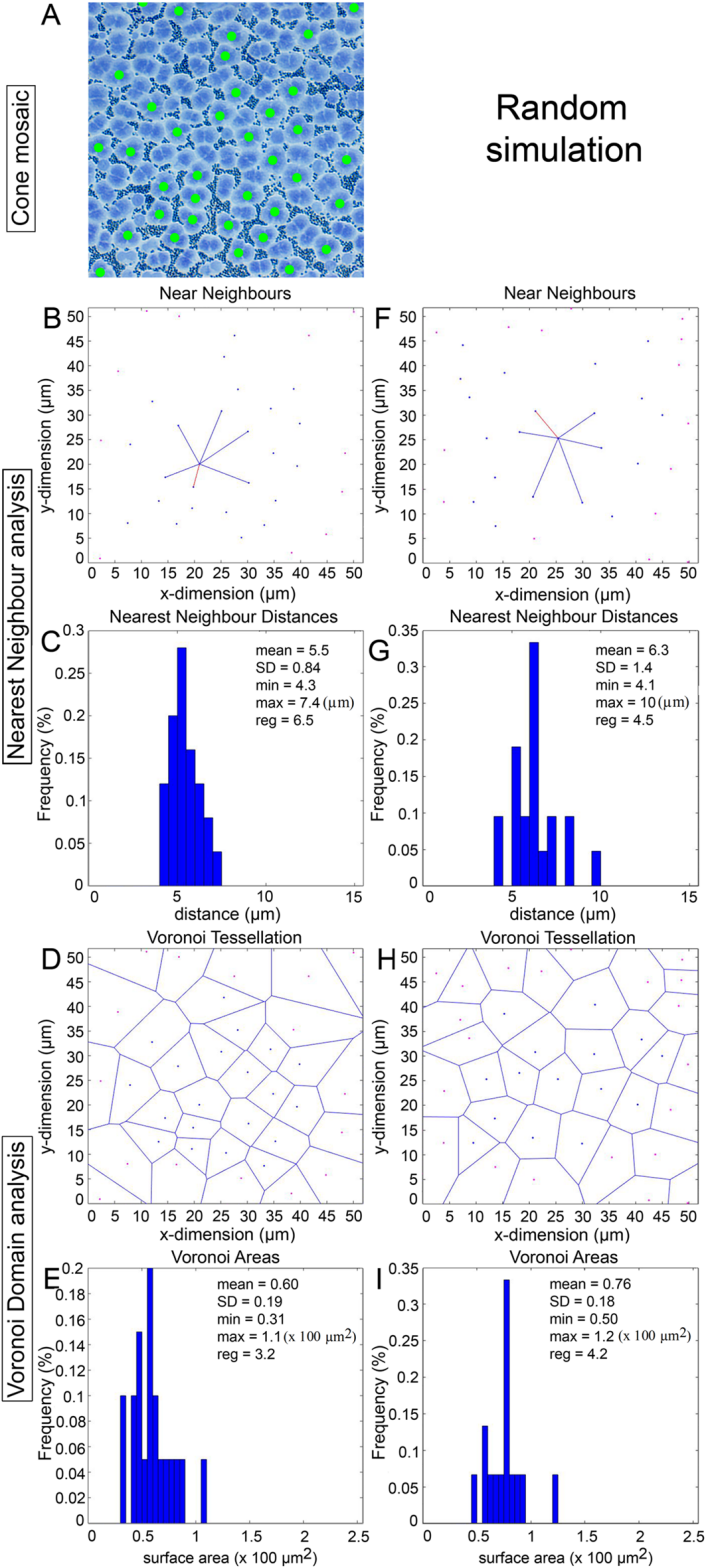
Spatial analysis of triple cone distributions (**A**) from the same mosaic shown in (**K**) of Fig. 9. Triple cones are marked with green dots. (**F**–**I**) Same presentation of data as per (**B**–**E**) but for a random distribution of points matched for density with that in (**A**) and constrained by triple cone soma size. Presentation of data as in Fig. 9.

Spatial analyses of the main cone distributions in the retina of Atlantic halibut undergoing metamorphosis revealed regularities that differed as a function of cone and mosaic type (Figs. 9, 10, 11). The greatest regularities were associated with lattice mosaics, which have characteristically high cone periodicity (Figs. 9A–J, 10A–E) and the lowest ones belonged to mosaics with triple cones (Figs. 9K–O, 10F–J, 11A–E). With the exception of cones forming the honeycomb mosaic, which were positioned side-by-side with minute spaces between them (Fig. 9A), single cones had greater nearest neighbour distances and Voronoi domain areas than their double or triple cone counterparts in the remaining mosaics (Figs. 9, 10, 11). This might have been expected from their lower relative densities (Figs. 9F, K, 10A, F, 11A).

To assess whether cones in non-lattice mosaics had some level of organization, their spatial statistics were compared to those of twenty random distributions of corresponding mean density and constrained by soma size (the latter was the mean diameter of 2.2 µm for the single cone, mean short axis distance of 3.0 µm for the double cone, and mean median of 4.1 µm for the triple cone; n = 30; Figs. 9P–S, 10K–N, 11F–I). None of the comparisons to the random distributions were significantly different, or the means were greater for the random distributions. Thus, all cone types (single, double, and triple) in mixed cone areas with high triple cone density (i.e., where the proportion of triple cones was statistically similar to that of double cones; Figs. 10F, 11A) were distributed randomly.

### Autocorrelation and density recovery profile analyses

**Figure 12:**
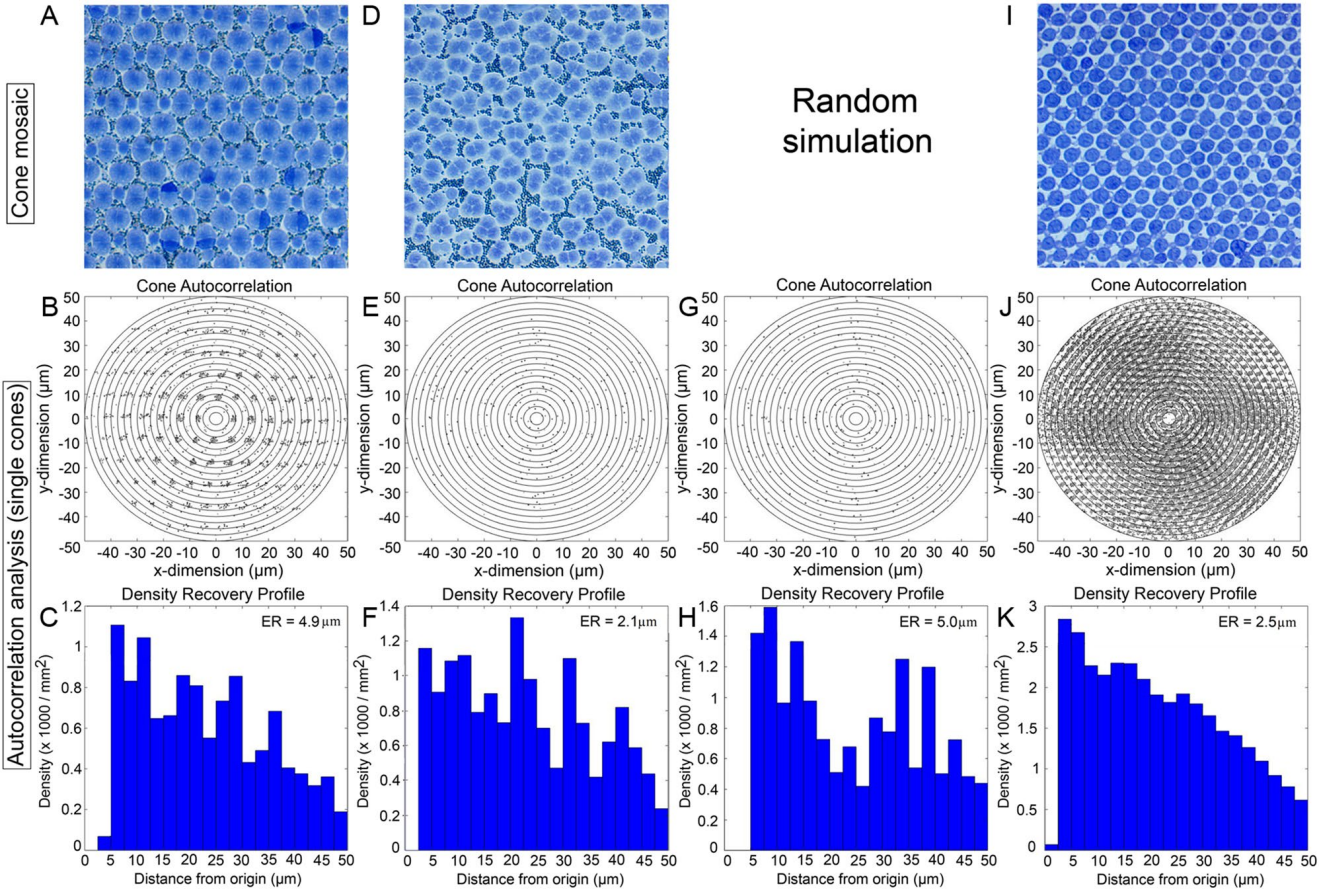
Autocorrelogram and density recovery profile for the single cone distributions (**A**–**F**, **I**–**K**) shown in Fig. 9, as well as the random distribution associated with the single cone in the triple cone area (**G**,**H**). The ER is the effective radius (or exclusion zone) which represents the size of the null region at the centre of the autocorrelogram.

**Figure 13:**
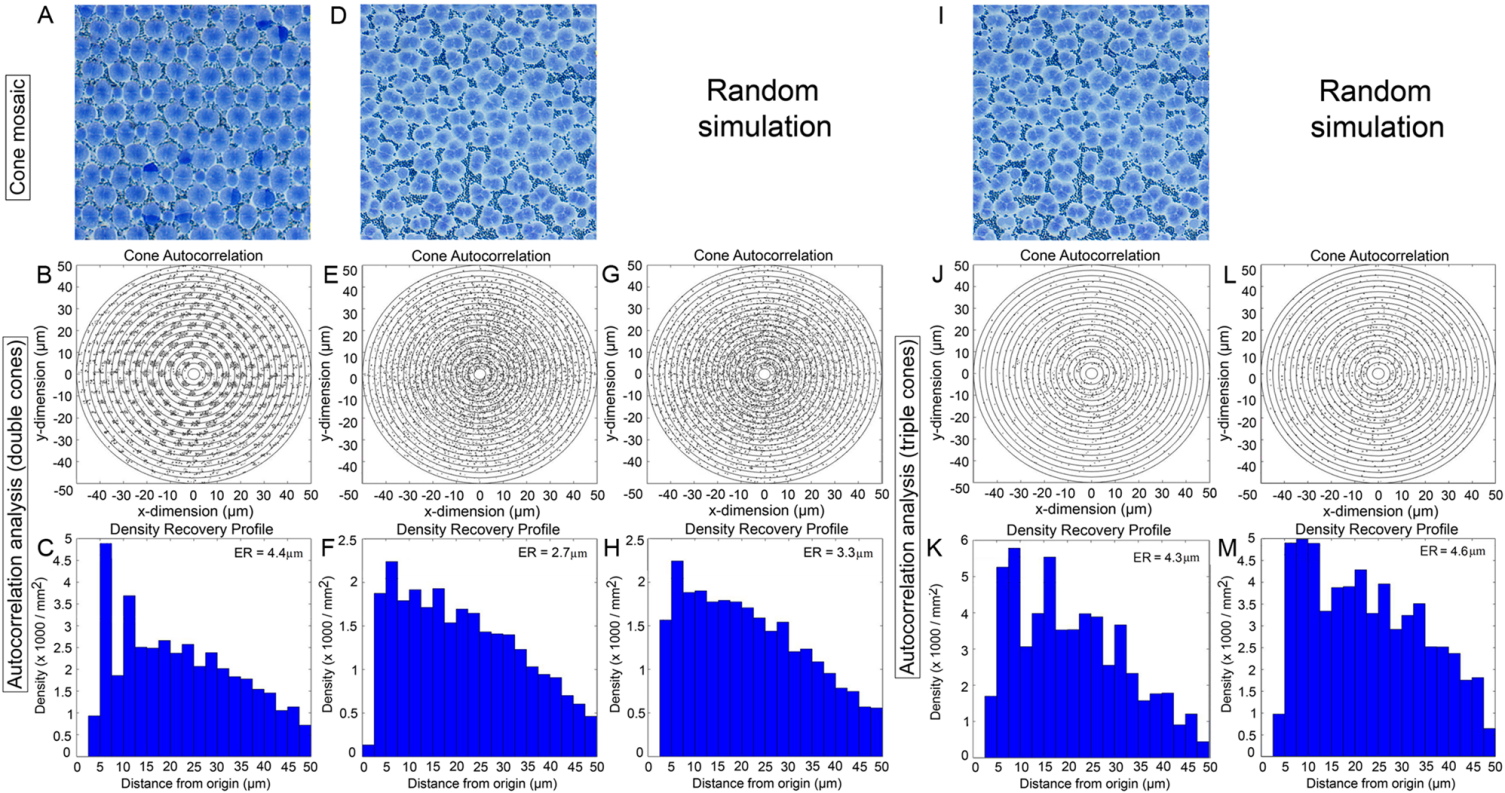
Autocorrelogram and density recovery profile for the double cone (**A**–**F**) and triple cone (**I**–**K**) distributions shown in Fig. 9, as well as the random distributions associated with the double cone (**G**,**H**) or triple cone (**L**,**M**) in the triple cone area. Presentation of data as per Fig. 12.

The autocorrelograms of morphological cone types that were part of square or honeycomb mosaics revealed a variety of lattice-like arrangements characterized by periodic foci with variable jitter (Figs. 12, 13). The density recovery profile, derived from the aurocorrelogram, showed a region surrounding the origin (the effective radius) where like-type cones were absent (Figs. 12B,C,J,K, 13B,C). This exclusion zone for single cones in the honeycomb mosaic was slightly greater than the diameter of a single cone (Fig. 12K). For double cones of the square mosaic, it approximated the size of the long axis of a double cone ellipsoid cross section (Fig. 13C). These results reflect the tight packing of these cone types in their respective lattices. The greatest distance between single cones (compared to double cones) in the square mosaic resulted in a larger effective radius for this cone type (Fig. 12C).

None of the autocorrelograms associated with cones in non-lattice distributions showed signs of periodicity (Figs. 12E, 13E,J). The density recovery profiles of cones in non-lattice mosaics (Figs. 12F, 13F,K) had effective radii that were statistically similar or lower than those obtained from simulations of random distributions (Figs. 12G,H, 13G,H,L,M).

### Genomic identification and phylogenetic analyses of Atlantic halibut opsins

Queries of the Atlantic halibut genome predicted eight opsin genes corresponding to five opsin classes: SWS1, SWS2, RH1, RH2, and LWS (Supplementary Table S1). Two genes were predicted for *rh2* and *sws2*, whereas one gene was predicted for each of the other opsin classes. Coding sequences corresponding to *rh2*, *sws2*, and *lws* were located on chromosome 5; sequences for *sws1* and *rh1* were located on chromosomes 23 and 7, respectively (Supplementary Fig. S2A–D).

Compared to the annotated genomes of turbot (*Scopthalmus maximus*) and zebrafish (*Danio rerio*), the positions of the predicted opsins and their flanking genes were in similar orientations (Supplementary Fig. S2A–D). Predicted *RH2* opsins were located in tandem and were flanked by *slc66a22.2* and *synaptoporin* (*synpr*) (Supplementary Fig. 2A). Initially, the annotated genome predicted three *rh2* coding sequences; however, one of these sequences (accession: XP_034442263.1) was incorrectly annotated as it had an unusual six exon structure and overlapped the regions of two other *rh2* sequences (Supplementary Table S1, Supplementary Fig. S3A). We hypothesized that this incorrect sequence corresponded to a *rh2* pseudogene, with only the first three exons being correctly annotated (Supplementary Fig. S3B), and did not consider it in further analyses.

The *sws2*-*lws* gene cluster on chromosome 5 was flanked by *hcfla* and *gnl3l*, consistent with similar genomic regions in turbot and zebrafish (Supplementary Fig. S2B). Within this cluster, two *sws2* genes were separated by a *sws2* pseudogene.

Previously reported Atlantic halibut opsins^11^ were used as queries in BLASTp analyses to confirm the classes of the predicted opsins (Supplementary Tables S2–S3). Sequence similarities in the range of 38 – 63% were observed between predicted opsins and those of a different class, whereas opsins of the same class were ≥ 80% identical (Supplementary Table S3. Predicted opsins with > 99% sequence similarity to opsins of their same class were determined to be the same as previously published opsins^11^. These opsins included SWS1 (100%), SWS2 (accession: XP_034440595.1) (99%), RH1 (100%), RH2 (accession: XP_034442261.1) (100%), and LWS (99%) (Supplementary Table S3). A lower percent identity for the other SWS2 (accession: XP_034441481.1) (75%) and RH2 (accession: XP_034442262.1) (86%) opsins suggested that these were novel opsins in Atlantic halibut.

To confirm that the predicted proteins were all visual opsins, key residues from these were compared to corresponding ones from visual opsins in zebrafish, and non-visual opsins in Atlantic halibut and zebrafish (Supplementary Table S4).

**Figure 14:**
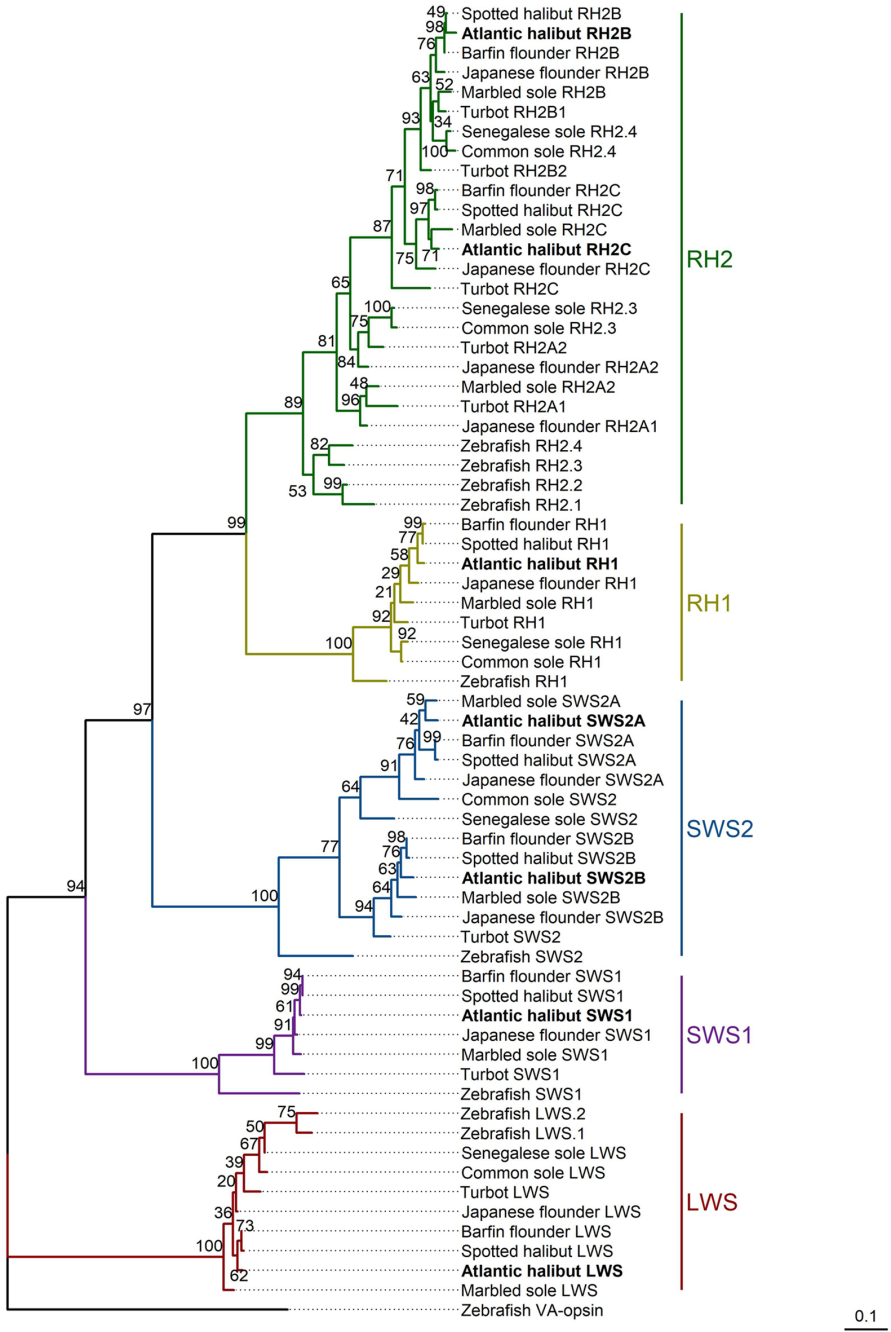
Maximum likelihood tree of flatfish and zebrafish amino acid sequences for five opsin classes: SWS1, SWS2, RH1, RH2, and LWS. Bootstrap values (*n* = 100) are located next to the branches and predicted opsins for Atlantic halibut are bolded. See Supplementary Table S11 for accession numbers used.

Based on phylogenetic analyses, Atlantic halibut opsins were classified as SWS1, SWS2A (accession: XP_034440595.1), SWS2B (accession: XP_034441481.1), RH1, RH2B (accession: XP_034442261.1), RH2C (accession: XP_034442262.1), and LWS and are referred to as such for the remainder of the manuscript (Fig. 14). No modifications at known amino acid tuning sites were found for SWS1, SWS2B, RH2B, or RH2C with respect to other flatfishes (Supplementary Tables S5–S9). An A292S amino acid substitution for Atlantic halibut SWS2A differed from other flatfishes (Supplementary Table S6). Marbled sole, *Pseudopleuronectes yokohamae*, and Atlantic halibut LWS share an A164S substitution (Fig. 14; Supplementary Table S9). Some heterogeneity across flatfishes was observed for RH1 sites 124 and 300; Atlantic halibut, spotted halibut (*Verasper variegatus*) and barfin flounder (*Verasper moseri*) all had a valine residue at site 300, but Atlantic halibut differed from these other two flatfishes with an A124G substitution. However, this A124G substitution does not impact the λ_max_ of the corresponding visual pigment^12^.

Except for SWS2A, the λ_max_ of reconstituted barfin flounder opsins^13^ corresponded well with some of our microspectrophotometry records of associated visual pigments and were therefore used as references for inferring the λ_max_ of Atlantic halibut opsins when bound to retinal as the chromophore. Therefore, the λ_max_ values of these opsins were predicted to be around 367 nm (SWS1), 416 nm (SWS2B), 494 nm (RH1), 506 nm (RH2B), and 490 nm (RH2C) (Supplementary Table S10). For SWS2A the corresponding opsin from Japanese flounder, *Paralichthys olivaceus*^14^, was used. The A292S substitution for SWS2A suggested a − 8 nm shift^15^ and a predicted λ_max_ near 458 nm. Based on the A164S substitution^15^, LWS was predicted to have a λ_max_ in the range 554–558 nm.

## Discussion

### Spatio-temporal dynamics of photoreceptor distributions

The retina of Atlantic halibut undergoing eye migration experienced major changes in photoreceptor types and distributions. The single cone type organized in honeycomb mosaic reported at the end of the yolk sac period (40 days post-hatching)^6^ started to reorganize to incorporate double cones^6^ as part of square mosaics (Fig. 2) toward the beginning of eye migration. The youngest halibut examined in this study, whose eye migration had barely started, had two regions of the retina, the centroventronasal and centrodorsotemporal, that contained double cones and square mosaics. At later developmental stages, the square mosaic began to appear in the centroventrotemporal retina and, by the end of eye migration, was present throughout the retina. This progression, which swept from ventronasal and dorsotemporal regions to ventrotemporal and, lastly, to the dorsonasal retina contrasts with a previous report stating that the start of the square mosaic was most advanced at the periphery near the *ora serrata* (at 70 days post hatching) and reached the centre of the retina by 100 days post-hatching^6^.

The seeming controversy between our study and previous work^6^ can be resolved by realizing that there are two independent mechanisms that create double cones and the square mosaic in the retinas of flatfishes^8^. One, common to all fishes, operates at the peripheral growth zone (*ora serrata*) through differentiation of progenitor cells into photoreceptors that become part of established (adult) mosaics, like the square mosaic^16^. The other appears to occur only among fishes with indirect development (i.e., species that undergo a pronounced metamorphosis where the larva and adult have very different form^17^). Here, there seems to be a re-arrangement of existing cones in central parts of the retina (present prior to the onset of metamorphosis^8^) that leads to square mosaic lattices with substantially more jitter than those forming directly at the periphery, as per our study. In other words, previous authors^6^ did not disambiguate between these two mechanisms leading to potential confusion over the progression of square mosaic formation in the non-peripheral retina prior to the start of metamorphosis.

The progression of double cone and square mosaic appearance reported here agrees with similar observations in winter flounder where two patches of retina, located in the ventronasal and dorsotemporal regions, were the first to express new opsin transcripts simultaneously (i.e., were present in all samples examined) from the start of metamorphosis^8^. As in our study, these authors observed that, at the start of eye migration, the ventronasal and dorsotemporal quadrants had double cones as part of emerging square mosaics whereas the dorsonasal and ventrotemporal quadrants had single cones in honeycomb formation^8^.

Another major discrepancy between our study and previous work^6^ pertains to the appearance of triple cones in the central retina as metamorphosis progressed. Upon completion of eye migration, these cones occupied a large area of central retina as part of random distributions along with double and single cones, whose distributions were also random. Triple cones have never been reported during metamorphosis of flatfishes^5–8^ but are present in the centrodorsal retina of several fish species during metamorphosis^18–20^. Some fishes, which are demersal or live in low light environments as adults, retain a high density of triple cones in a restricted area of centrodorsotemporal retina^21^ often located next to a fovea^22^. Other, nocturnal fishes, like some species of holocentrids, also have triple cones. They are primarily located in regions of high cone density, along a central meridian, but constitute less than 0.5% of the total cone population^23^.

Based on the appearance of triple cones around the time when double cones and square mosaics start forming in the retinas of some marine fishes, it has been hypothesized that triple cones may be part of a transitional mosaic between the larval honeycomb mosaic and the adult square mosaic^18^. Two observations suggest otherwise, however. First, in Atlantic halibut, triple cones were rare in the ventral retina and scarce in the nasal compared to the temporal retina. If triple cones were part of a necessary transitional mosaic leading to the square mosaic, then the ventral and nasal regions of the central retina would not have developed such a mosaic or, at least, not fully. Second, triple cones are retained in the centrodorsotemporal retina of adults of other fish species^21,22^ suggesting a functional role within the random mosaics that they are a part of.

### Different square mosaic formation processes in the central and peripheral retina

Our results support two different mechanisms of square mosaic formation in the retina of Atlantic halibut during metamorphosis. At 720 ATU, the centrodorsonasal and centroventrotemporal areas had only singles cones and these were arranged in honeycomb formation. Because a square mosaic was present in these regions of the retina by the end of metamorphosis, and no new cones are known to differentiate in the main retina of flatfishes^8^, existing single cones must have coalesced to form the square mosaic (a conclusion previously reached for the winter flounder by other authors^5,8^). Electron microscopy evidence suggesting such cone fusion has been provided for the black bream, *Acanthopagrus butcheri*, and the West Australian dhufish, *Glaucosoma hebraicum*, two marine fishes that also undergo metamorphosis^18,19^.

Near the periphery, the regularity of the square lattice contrasted with the jitter present in multiple square mosaic regions of the central retina. At the growth zone, the square mosaic contained corner cones (Supplementary Fig. S4), and these disappeared with distance from the periphery, as has been shown for the Atlantic salmon, *Salmo salar*^16^. These observations suggest a direct path of square mosaic formation, as established for other fishes.

### Topographical changes in visual acuity match foraging ecology

In the non-peripheral retina, spatial resolving power, our theoretical measure of visual acuity, was greatest in the ventrotemporal retina of the youngest Atlantic halibut examined. At the end of metamorphosis, the area of highest resolving power had shifted to the dorsotemporal retina. The values computed were nearly identical to those published for the plaice, *Pleuronectes platessa*, another flatfish, where spatial resolving power (independently derived from measures of radially-oriented central retina) was ~ 0.5º at the start of metamorphosis and ~ 0.3º by the end^24^.

The shift in area of greatest resolving power conforms with the foraging needs of the fish as it transforms from a bilaterally symmetric larva to a flattened, demersal juvenile. Pelagic fishes, including larvae, often have the highest cone densities in the ventrotemporal retina and strike at prey that contrast against the water background located in front of them or slightly above^25–27^. Demersal fishes, on the other hand, often strike at prey that contrasts against the bottom of the water body^28^. As such, the observed shift in area of greatest resolving power would be expected to maximize prey capture, the strongest determinant of fish growth and survival.

### A potential role for the triple cone in achromatic target detection

That the highest visual acuity in the postmetamorphic fish coincided with the area of densest triple cone presence suggests that the random mosaic associated with triple cones is not an unintentional by-product of development but serves a specific function. The adults of other demersal fishes have a similar triple cone area in the centrodorsotemporal retina, in the vicinity of where the highest cone densities are found^21,22^. Spatial analyses of flatfish mosaics show that greater cone packing can be achieved through a combination of different cone morphologies (including the triple cone) compared to that from the square mosaic^21^. It has been argued, however, that a regular mosaic should provide better chromatic visual acuity due to the regular distribution of spectral inputs across the visual field^21,29,30^. For a fish that lives in habitats with low light, visual acuity based on chromatic contrast may not be as important as improving photon catch and, potentially, achromatic detection of targets.

In the random mosaic region, the ratio of the sum of triple and double cones to single cones is the same (~ 2) as that of double to single cones in nearby square mosaic regions. As such, the total ratio of longer to shorter wavelength input should be similar regardless of the mosaic, the major difference being the regularity of chromatic input. Based on these observations and the greater cone packing achievable with the random mosaic^21^, we propose that triple cones serve an achromatic area of high visual acuity. Because of the ventral position of the optic nerve head, the area of random mosaic appears to view the forward horizon. It may therefore represent a specialized region for the early detection of large groundfish predators, like many species of elasmobranchs, that cruise the ocean floor in search of demersal prey. Such predators lack significant colour and would be readily detected achromatically by improving photon catch. Interestingly, photoreceptors in this area also have among the longest outer segments, further improving photon catch.

### Visual pigment co-expression: a potential shift toward longer wavelength absorbance

Absorbance measurements from postmetamorphic retinas indicated the presence of at least two opsins, with associated visual pigments peaking at 431 nm and 457 nm, among the single cone population. The λ_max_ and bandwidth at half maximum of the rod visual pigment (mean ± SD = 4170 ± 234 cm^-1^, n = 20) suggested retinal (the aldehyde of vitamin A_1_) as the only chromophore in the retina^31^, as has been measured by High Performance Liquid Chromatography in starry flounder, *Platichthys stellatus*^32,33^. Co-expression of the S(431) pigment with another visual pigment [potentially the M(500) or M(514)] in a given single cone was common and may illustrate a progressive switch toward the longer wavelength-associated opsin as the juvenile moves to greater depths. A progression toward longer wavelength-absorbing visual pigments occurs in multiple cone types of the juvenile starry flounder^33^.

### Visual opsin repertoire of Atlantic halibut

The predicted proteins from the Atlantic halibut genome provided evidence for six visual opsin genes. Phylogenetic and tuning site analyses revealed that these opsins correspond to SWS1, SWS2A, SWS2B, RH1, RH2B, RH2C, and LWS. This expands the previous opsin repertoire known for Atlantic halibut which included SWS1, SWS2, RH1, RH2, and LWS^11^. The updated opsin repertoire is consistent with those of other flatfish species, with six visual opsins reported for the common sole and the Senegalese sole^21^, eight for starry flounder^32^ and marbled sole, *Pseudopleuronectes yokohamae*^34^, and nine for turbot, *Scophthalmus maximus*^35^. In particular, expansion of the *rh2* repertoire in Atlantic halibut follows findings in other flatfishes where *rh2b* and *rh2c* genes are common and *rh2a* is often absent or present as a pseudogene ^13,14,21,34,35^. Tandem duplications of *sws2* genes are also commonly found in flatfishes^13,14,34^ and other teleosts^36^.

Both the number of predicted opsins and the surmised λ_max_ of their visual pigments when bound to retinal only partially matched microspectrophotometric records. The predicted SWS2A opsin matched the S(457) visual pigment, RH1 corresponded to the rod(491) visual pigment, and the LWS opsin matched the L(550) visual pigment. The S and M pigments found by microspectrophotometry were not a good match for the SWS2B or RH2 opsins. Previous work comparing the λ_max_ of M visual pigments in the starry flounder^32^ and the common sole^21^ to those of barfin flounder^13^ or marbled sole^34^ (the latter two sets measured from opsin-retinal reconstitutions or predicted from opsin tuning sites, respectively) showed that the *in-vivo* measured pigments were significantly long wavelength shifted with respect to *in-vitro* determinations or predictions. With some opsins, this was the case despite having identical amino acids at all tuning sites between species^13,21^. These results suggest that alternative tuning sites or interactions between multiple tuning sites play important roles in determining the λ_max_ of S and M visual pigments in flatfishes.

## Conclusion

Retinal transformation at metamorphosis in Atlantic halibut is a complex process that prepares the animal for the visual ecology demands of a demersal life style. Photoreceptors organization changes from a honeycomb mosaic consisting of single cones to two novel formations: the square mosaic, which occupies most of the post-metamorphic retina, and a random mosaic consisting of single, double and triple cones located in a restricted region of the dorsotemporal retina. In parallel, a square mosaic of greater regularity forms directly from progenitor cells in the peripheral growth zone, and both mosaics coalesce during metamorphosis. The central region of greatest visual acuity shifts from the ventrotemporal to the dorsotemporal retina in accordance with the change in foraging behaviour that accompanies the transition from pelagic to benthic life style. The retina has the potential for colour vision containing a minimum of six cone visual pigments and one rod visual pigment. Such composition resembles that of other flatfishes that have been studied which show abundant expression of multiple S and M visual pigments, but diminished expression of UV and L visual pigments with age.

## Materials and methods

### Fish husbandry and collections

Atlantic halibut were reared from the fertilized egg in 100 L recirculating saltwater indoor tanks at a temperature of 7.5  ± 1.6 °C and maintained under artificial illumination (λ range: 350–750 nm, irradiance: 1.2 × 10^15^ photons m^-2^ s^-1^) provided by tungsten-halogen tubes (Scotian Halibut Ltd, Nova Scotia, Canada). Peak hatching occurred at 14 days post-fertilization [~  90 Accumulated Temperature Units (ATUs), calculated as the product of days from fertilization and mean water temperature]. Hatched larvae had a prominent yolk sac which was fully absorbed around 50 days post-hatching (~  320 ATUs). Pre-metamorphic larvae were fed a combination of freeze-dried Gemma Diamond 0.5 and 0.8 mm pellets (Skretting), added every 30 min throughout the day at a density of 2 pellets per fish, and *Artemia* nauplii, added twice a day at a density of 10–15 metanauplii ml^−1^ day^−1^. Fish nearing complete metamorphosis, or having completed it, were fed a mixture of Gemma Diamond 0.8, 1.0 and 1.2 mm every hour at a density of 3 pellets per fish and *Artemia* nauplii twice a day at a density of 5 to 10 metanauplii ml^−1^ day^−1^. Clay was added to the tanks every day to recreate the turbid water conditions found in nature.

Collections of specimens took place at 82, 89, 96 and 103 days post-hatching, corresponding to 720, 773, 825 and 878 ATU. At each stage, fish were collected in the light-adapted state for *in-situ* hybridization analyses and, separately, for morphological observations. At 720 ATU, eye migration had just started in the majority of fish examined and, by 878 ATU, metamorphosis was complete (Fig. 1). In addition to these collections at the aquaculture facility, post-metamorphic juvenile fish were flown live to the University of Victoria aquatic facility (British Columbia, Canada) for measurements of visual pigment absorbance by microspectrophotometry. The study is reported in accordance with ARRIVE guidelines.

### Histology

Fish were euthanized and the entire bodies fixed in primary fixative (2.5% glutaraldehyde, 1% paraformaldehyde in 0.08 M PBS, pH = 7.4) at 4ºC for a minimum of 72 h. The retina from the migrating and non-migrating eye were individually extracted, rinsed in 0.08 M PBS, and cut into four quadrants (ventrotemporal, VT; ventronasal, VN; dorsonasal, DN; and dorsotemporal, DT). The quadrants were post-fixed in secondary fixative (1% osmium tetroxide in 0.08 M PBS) for 1 h at 4ºC. Following a brief rinse in distilled water, the tissue was dehydrated through a series of solutions of increasing ethanol concentration, infiltrated with mixtures of propylene oxide and EPON resin, and embedded in 100% EPON resin. Retinal blocks were cut tangentially, in 1–2 µm steps, and stained with Richardson’s solution (1:1 mixture of 1% Azure II in dH_2_O and 1% Methylene blue in 1% NaB_4_O_7_ ) to reveal the cone mosaic^23,31^. Digital images of sections were acquired with an E-600 Nikon microscope equipped with a DXM-100 digital camera. Photographs were obtained using a Plan/Apo 60X/1.40 objective and further amplified 1.5 × with an optical tourette on the microscope (total magnification 90x). Observations of mosaic types in different sectors were carried out on 10 retinas (5 each from migrating and non-migrating eyes). From these, four retinas with the largest tangential mosaic area (0.0106 mm^-2^) spanning the region of widest cone ellipsoid cross sections to the bases of the outer segments were selected for density determinations and spatial analyses.

### *In-situ* hybridization

These experiments were undertaken on 10 retinas to verify morphological observations of rod photoreceptor appearance using riboprobes. Whole bodies of euthanized fish were fixed (4% paraformaldehyde in 0.08 M PBS, pH 7.4) at 4 °C for a minimum of 72 h. For a given fish, the head was processed as a whole or the eyes were extracted from it and processed individually. Following several rinses in 0.08 M PBS, the tissue was cryoprotected in sucrose solution (25% sucrose, 20% optimal cutting temperature [OCT] medium in 0.08 M PBS) and embedded in 100% OCT medium (Cedar Lane Laboratories). The resulting blocks were cryosectioned in 7–10 µm steps in either tangential (for individual eyes) or coronal (for heads) planes to reveal the cone mosaic or photoreceptors along their lengths in the medial retina, respectively. Serial sections were deposited cyclically on poly-L-lysine coated slides and hybridized with species-specific riboprobes against the *rh1* and *rh2* opsin mRNAs of Atlantic halibut^9,11^.

The *rh2* and *rh1* riboprobes were 591 and 447 bp long, respectively. The primers were as follows: *rh2* forward 5’-GCTCAATAGAAACAACAGAC-3’, *rh2* reverse 5’-GCCAGGACGACCAGAGACCA-3’; *rh1* forward 5’-CTCAACAGCCAGAAGAAACA-3’; *rh1* reverse 5’-GTAACGAGACCAGCCAACAA-3’. Each probe showed 70% similarity to the non-corresponding opsin.

In situ hybridizations were performed according to procedures detailed in the literature^37,38^. Briefly, this involved rehydrating the sections, permeabilizing them in 10 µg/ml proteinase K for 5 min, exposure to 0.1 M triethanolamine containing 0.25% acetic anhydride, dehydration, and overnight hybridization at 56 °C with 4 µg of cRNA probe in 50% formamide, dextran sulfate and goat serum. Following immunodetection with anti-Digoxigenin F_ab_ fragments conjugated to alkaline phosphatase (1:3000) for 2 h at room temperature, the riboprobe was visualized with NBT/ BCIP substrate^38^. Cross hybridization of a riboprobe with the non-corresponding opsin did not take place as evidenced by the different level of label of photoreceptor myoids in the light-adapted retina (corresponding to cones and rods; Fig. 6).

Sections were viewed with the E-600 Nikon microscope equipped for DIC imaging. Photographs were obtained with the DXM-100 digital camera at 90 × magnification using the 60x/1.40 objective and microscope tourette.

### Morphometric analyses

Digital micrographs of tangential sections, each covering a 52 × 52 µm^2^ area, were analyzed with Photoshop (Adobe Systems) to extract the X–Y coordinates (centroids) of every morphological cone type in the field of view. The coordinates were then imported into a customized Matlab program that computed the Delauney tessellation of the field, from which the nearest neighbour distance of each individual cell was determined, as was their Voronoi domain area (i.e., the area surrounding each cell that encloses the territory closer to that cell than to any of the neighbours)^39^. Each analysis excluded “border cells”, i.e., those with uncertain nearest neighbour distances or Voronoi domain areas. In each case, the regularity index was defined as the mean divided by the standard deviation.

To assess whether the cone distributions analyzed had higher order (lattice-like) periodicity, a spatial auto-correlation analysis was carried out that examined the positioning of each cell with respect to all other cells across the mosaic. The Density Recovery Profile was derived from each autocorrelogram, providing a plot of the mean density of cells as a function of distance from each cell. The autocorrelogram permits the detection of higher order patterning and the Density Recovery Profile provides a measure of the exclusion zone surrounding each cell where other homotypic cells are less likely to be found than at further distances^39^.

To assess whether cell distributions with low regularity indices were different from random distributions, their spatial statistics were compared to those of simulated random fields of cells matched for density and constrained by soma size. Non-random retinal mosaics of cells show greater regularity indices and exclusion zones to those from corresponding random simulations resulting in effective radii that are larger than if determined by soma size alone^40^.

Statistical analyses to assess differences in mosaic regularity and cone density between retinal sectors were based on analysis of variance (ANOVA) with post-hoc grouping tests (Student-Neuman-Keuls, Tukey HSD) evaluated at α = 0.05 level of significance.

### Visual acuity

Theoretical determinations of spatial resolving power (in degrees of arc) were calculated as the inverse of the Nyquist frequency (f_N_) (in cycles per degree) using the equation:

$${\text{f}}_{{\text{N}}} = (\pi *{\text{PND}}/{36}0{^\circ })*\left( {{2}*{\text{D}}/\left( {3} \right)^{{{1}/{2}}} } \right)^{{{1}/{2}}}$$

where D is the photoreceptor cell density (in mm^-2^) and PND is the posterior nodal distance (in mm)^22^. PND was approximated as the product of lens radius and 2.55 (the mean Matthiessen’s ratio for teleost eyes^41^). The photoreceptor cell density was computed from the sum of all cone types measured in a 0.0106 mm^-2^ area, with each double cone and triple cone contributing two and three cells, respectively. Based on the resolving power, the distance at which a given region of the retina would detect a 0.5 mm size food item, such as the typical food pellet encountered in aquaculture conditions or prey copepod consumed in nature, was computed.

### Microspectrophotometry

This analysis was performed on four post-metamorphic juveniles with mean weight ± SD and total length ± SD of 3.6 ± 0.8 g and 7.5 ± 0.5 cm, respectively. Individual fish were dark adapted for 8 h, following which the fish was euthanized, and the retina extracted under infrared illumination. Small pieces of retina were teased apart and prepared for viewing with the dichroic microspectrophotometer (DMSP) as per previous studies^42–44^. The DMSP is a computer-controlled, wavelength-scanning, single-beam photometer that simultaneously records average and polarized transmitted light fluxes through microscopic samples^42,44^. The DMSP was equipped with ultrafluar (Zeiss) objectives: 32/0.4 for the condenser and 100/1.20 for the objective. With the aid of reference measurements recorded through cell-free areas, individual photoreceptor outer segments were illuminated sideways with a measuring beam of rectangular cross section of ca. 2 × 0.6 μm. Absolute absorbance spectra were computed in 2 nm increments from the obtained transmittances (each spectrum consisted of an average of 8 scans). The solid spectra (fits) were derived from experimental data by Fourier filtering^43^.

### Analysis of spectra consisting of multiple visual pigments

Because the S(431) absorbance spectrum showed a “hump” in the range 450–550 nm, suggesting the presence of multiple visual pigments, the Simplex algorithm^45^ was used to fit the data with a combination of visual pigment absorbance curves using the equation^46^:

$${\text{R }} = \, \left[ {{\text{k}}_{{\text{i}}} \,{\text{A}}_{{\text{i}}}^{{\text{p}}} \left( \lambda \right)} \right]^{{{1}/{\text{p}}}}$$

where R is the response (overall absorbance) function, A_i_^p^ is the absorbance of pigment i at light of wavelength λ, and p is an exponent resulting from the mathematical requirement that the absorbance function to be fitted be differentiable at the origin^46^. The variables k_i_ are coupling constants resulting from the best fit of the model to the data. The visual pigment absorbance values used in the model were generated with an eighth-order polynomial template^47^ based on the λ_max_ of visual pigments measured by microspectrophotometry. The use of templates ensures β-band representation of individual visual pigments, which is not always present in microspectrophotometry records.

### Genomic analyses

Zebrafish visual opsins (Supplementary Table S11) were used as query sequences to search predicted proteins from the reference genome of Atlantic halibut^48^ (NCBI *Hippoglossus hippoglossus* Annotation Release 100; GCF_009819705.1) using BLASTp (v.2.12.0). Predicted proteins with e-values < 1e-5 and percent identities ≥ 50% were retained. Duplicated results were filtered by accession number using R (v.4.1.0)^49^ and RStudio (v.1.4.1717)^50^. Locations of the corresponding coding sequences and their immediate flanking genes were compared to those in the reference genomes of turbot (NCBI *Scophthalmus maximus* Annotation Release 100; GCF_013347765.1)^51^ and zebrafish (NCBI *Danio rerio*, GRCz11, NCBI Annotation Release 106; GCF_000002035.6) and used to further narrow down the predicted opsins using the NCBI Genome Browser and Gene Graphics^52^. Coding sequences that were eliminated from further analysis can be found in Supplementary Table S12 with the corresponding justification.

To confirm the assigned opsin classes of the predicted proteins, opsin sequences reported previously^11^ were used to query the subset of predicted Atlantic halibut proteins using BLASTp, and sequences with an e-value < 1e-5 and a percent identity ≥ 70% retained. To confirm that the predicted proteins were visual opsins, a multiple sequence alignment was performed that incorporated visual zebrafish opsins and non-visual zebrafish and Atlantic halibut opsins (Supplementary Table S4). Visual opsins were distinguished from non-visual opsins by their identity at key amino acid sites^53^. All multiple alignments were performed and edited using ClustalX (v.2.0)^54^ and Qiagen CLC Sequence Viewer (v.8.0) (www.qiagenbioinformatics.com). Amino acid residue numbers were standardized to bovine rhodopsin (Accession: NP_001014890.1)^55^.

The predicted Atlantic halibut opsins were assigned to opsin subclasses using phylogenetic analyses and tuning sites from key amino acid residues for each of the visual opsins^12,35,56,57^ . Phylogenetic trees were constructed with MEGAX^58^ using maximum likelihood and boostrap analyses (n = 100) and visualized using R and ggtree^49,50,59^. Sequences used in these analyses (Supplementary Tables S11, S13) were obtained from GenBank (https://www-ncbi-nlm-nih-gov.proxy.lib.sfu.ca/genbank) and SoleaDB^60^, with zebrafish VA opsins used as the outgroup.

### Animal experimentation approval

All animal use was approved by the Animal Care committees of Simon Fraser University (protocol # 1126B-10) and the University of Victoria (protocol # 2017–005), which abide by regulations set by the Canadian Council for Animal Care.

## Supplementary Information

Supplementary Information.

## Data Availability

Data are available in the manuscript and supplementary materials. Anything further is available from the authors upon reasonable request.
