# Author Correction: Photoreceptor distributions, visual pigments and the opsin repertoire of Atlantic halibut (*Hippoglossus hippoglossus*)

**DOI:** 10.1038/s41598-022-19236-y

**Published:** 2022-09-06

**Authors:** Kennedy Bolstad, Iñigo Novales Flamarique

**Affiliations:** 1 Department of Biological Sciences, Simon Fraser University, Burnaby, BC V5A 1S6 Canada; 2 Department of Biology, University of Victoria, Victoria, BC V8W 2Y2 Canada

Correction to: *Scientific Reports* 10.1038/s41598-022-11998-9, published online 16 May 2022

The original version of this Article contained repeated errors. Due to an error in the mean rearing temperature, all ATUs (Accumulated Temperature Units) were stated incorrectly.

As a result, in the Results section, under the subheading ‘Eye morphology and migration’,

“The youngest Atlantic halibut examined (96 days post-fertilization or 960 ATU; ATU are the Accumulated Temperature Units, i.e. the product of mean rearing water temperature (10 °C) and days from fertilization, 96) were bilaterally quasi-symmetrical with a compressed, oval shaped body and similar pigmentation on either side of the body (Fig. 1A,E).

now reads:

“The youngest Atlantic halibut examined (96 days post-fertilization or 720 ATU; ATU are the Accumulated Temperature Units, i.e. the product of mean rearing water temperature (7.5 °C) and days from fertilization, 96) were bilaterally quasi-symmetrical with a compressed, oval shaped body and similar pigmentation on either side of the body (Fig. 1A,E).”

And,

“Migration of the eye proceeded throughout the study period (Fig. 1A–D) such that between 1030 and 1100 ATU (i.e., 103 and 110 days post-fertilization, respectively) the migrating eye had reached the mid-line (Fig. 1C,F) or proceeded beyond, to the other side of the head. The oldest halibut examined (1170 ATU, or 117 days post-fertilization) had both eyes on the same side of the head (the ocular side, Fig. 1D,G) whereas the opposite side (the blind side) rested on the bottom and had reduced pigmentation.”

now reads:

“Migration of the eye proceeded throughout the study period (Fig. 1A–D) such that between 773 and 825 ATU (i.e., 103 and 110 days post-fertilization, respectively) the migrating eye had reached the mid-line (Fig. 1C,F) or proceeded beyond, to the other side of the head. The oldest halibut examined (878 ATU, or 117 days post-fertilization) had both eyes on the same side of the head (the ocular side, Fig. 1D,G) whereas the opposite side (the blind side) rested on the bottom and had reduced pigmentation.”

Under the subheading ‘Photoreceptor types and mosaics’,

“At 960 ATU, the retina had two main types of cone photoreceptors: single and double cones (Fig. 2A,C,E,G).”

now reads:

“At 720 ATU, the retina had two main types of cone photoreceptors: single and double cones (Fig. 2A,C,E,G).”

And,

“At 1030 ATU, double cones were present in the centroventrotemporal retina forming a square mosaic region of varying disposition (Fig. 2D).”

now reads:

“At 773 ATU, double cones were present in the centroventrotemporal retina forming a square mosaic region of varying disposition (Fig. 2D).”

“By the time eye migration was complete (1170 ATU), the honeycomb mosaic was absent from the retina and other cone formations had emerged (Figs. 3, 4 and 5).”

now reads:

“By the time eye migration was complete (878 ATU), the honeycomb mosaic was absent from the retina and other cone formations had emerged (Figs. 3, 4 and 5).”

“As per the trends in the dorsonasal retina, the ventral retina at 1170 ATU consisted of square mosaics with variable jitter in the lattice (Fig. 5A,C,D; white rectangles and circle).”

now reads:

“As per the trends in the dorsonasal retina, the ventral retina at 878 ATU consisted of square mosaics with variable jitter in the lattice (Fig. 5A,C,D; white rectangles and circle).”

And,

“At all locations, whether characterized by the honeycomb mosaic or by the presence of double cones, the retina of Atlantic halibut at 960 ATU labelled prominently with the *rh2* riboprobe along the inner segment myoids of cones (Fig. 6A,B; green arrows). In contrast, the *rh1* riboprobe failed to label cryosections characterized by a honeycomb mosaic (Fig. 6C,D). At 1170 ATU, both the *rh2* and *rh1* riboprobes labelled, respectively, the myoid region of double cone members (Fig. 6E) and those of rods (Fig. 6F).”

now reads:

“At all locations, whether characterized by the honeycomb mosaic or by the presence of double cones, the retina of Atlantic halibut at 720 ATU labelled prominently with the *rh2* riboprobe along the inner segment myoids of cones (Fig. 6A,B; green arrows). In contrast, the *rh1* riboprobe failed to label cryosections characterized by a honeycomb mosaic (Fig. 6C,D). At 878 ATU, both the *rh2* and *rh1* riboprobes labelled, respectively, the myoid region of double cone members (Fig. 6E) and those of rods (Fig. 6F).”

Under the subheading ‘Cone densities and measures of visual acuity’,

“A summary topographical schematic showing mean cone densities in the Atlantic halibut retina at the two extreme developmental time periods examined (960 and 1170 ATU) revealed profound retinal transformations (Fig. 7). At 960 ATU, the greatest cone density within the central retina was found in the ventrotemporal quadrant and the lowest in the dorsotemporal quadrant (Fig. 7A).”

now reads:

“A summary topographical schematic showing mean cone densities in the Atlantic halibut retina at the two extreme developmental time periods examined (720 and 878 ATU) revealed profound retinal transformations (Fig. 7). At 720 ATU, the greatest cone density within the central retina was found in the ventrotemporal quadrant and the lowest in the dorsotemporal quadrant (Fig. 7A).”

And,

“At 1170 ATU, the region of the central retina with the greatest cone density had shifted to the dorsotemporal quadrant (Fig. 7C). This region, with theoretical resolving power similar to that of the peripheral retina, also had the greatest density of triple cones (Fig. 7D). Despite an overall reduction in cone density in the central retina during development from 960 to 1170 ATU (Fig. 7A,C), theoretical resolving power generally improved (Fig. 7B,D) as a result of increased lens size.”

now reads:

“At 878 ATU, the region of the central retina with the greatest cone density had shifted to the dorsotemporal quadrant (Fig. 7C). This region, with theoretical resolving power similar to that of the peripheral retina, also had the greatest density of triple cones (Fig. 7D). Despite an overall reduction in cone density in the central retina during development from 720 to 878 ATU (Fig. 7A,C), theoretical resolving power generally improved (Fig. 7B,D) as a result of increased lens size.”

Furthermore, in the Discussion section, under the subheading ‘Different square mosaic formation processes in the central and peripheral retina’,

“At 960 ATU, the centrodorsonasal and centroventrotemporal areas had only singles cones and these were arranged in honeycomb formation.”

now reads:

“At 720 ATU, the centrodorsonasal and centroventrotemporal areas had only singles cones and these were arranged in honeycomb formation.”

In the Materials and methods section, under the subheading ‘Fish husbandry and collections’,

“Atlantic halibut were reared from the fertilized egg in 100 L recirculating saltwater indoor tanks at a temperature of 10 ± 1.6 °C and maintained under artificial illumination (λ range: 350–750 nm, irradiance: 1.2 × 10^15^ photons m^−2^ s^−1^) provided by tungsten-halogen tubes (Scotian Halibut Ltd, Nova Scotia, Canada). Peak hatching occurred at 14 days post-fertilization [~ 140 Accumulated Temperature Units (ATUs), calculated as the product of days from fertilization and mean water temperature]. Hatched larvae had a prominent yolk sac which was fully absorbed around 60 days post-hatching (~ 740 ATUs).”

now reads:

“Atlantic halibut were reared from the fertilized egg in 100 L recirculating saltwater indoor tanks at a temperature of 7.5 ± 1.6 °C and maintained under artificial illumination (λ range: 350–750 nm, irradiance: 1.2 × 10^15^ photons m^−2^ s^−1^) provided by tungsten-halogen tubes (Scotian Halibut Ltd, Nova Scotia, Canada). Peak hatching occurred at 14 days post-fertilization [~ 90 Accumulated Temperature Units (ATUs), calculated as the product of days from fertilization and mean water temperature]. Hatched larvae had a prominent yolk sac which was fully absorbed around 50 days post-hatching (~ 320 ATUs).”

And,

“Collections of specimens took place at 82, 89, 96 and 103 days post-hatching, corresponding to 960, 1030, 1100 and 1170 ATU. At each stage, fish were collected in the light-adapted state for in-situ hybridization analyses and, separately, for morphological observations. At 960 ATU, eye migration had just started in the majority of fish examined and, by 1170 ATU, metamorphosis was complete (Fig. 1).”

now reads:

“Collections of specimens took place at 82, 89, 96 and 103 days post-hatching, corresponding to 720, 773, 825 and 878 ATU. At each stage, fish were collected in the light-adapted state for in-situ hybridization analyses and, separately, for morphological observations. At 720 ATU, eye migration had just started in the majority of fish examined and, by 878 ATU, metamorphosis was complete (Fig. 1).”

Consequently, Figures 1, 2, 6 and 7 and their respective legends contained errors. The original Figures 1, 2, 6 and 7 and accompanying legends appear below.

Finally, the legends of Figures 3, 4, 5 and 9 contained repeated errors, where

“Micrographs of tangential EPON sections showing cone distributions from the dorsotemporal retinal quadrant at 1170 ATU.”

now reads:

“Micrographs of tangential EPON sections showing cone distributions from the dorsotemporal retinal quadrant at 878 ATU.”

And,

“Spatial analysis of single cone distributions from representative mosaics in the retina of Atlantic halibut undergoing metamorphosis. Single cones are marked with yellow dots. **(A–E)** Cone mosaic from the centrodorsonasal retina at 1030 ATU **(A)**; Nearest neighbour analysis of double cone centroids, illustrating the near neighbours of a single cone, including its nearest neighbour in red **(B)** and their frequency distribution **(C)** [statistics (in µm) are the mean nearest neighbour distance, its standard deviation (SD), the minimum (min) and maximum (max) nearest neighbour distances, and the regularity index=mean/SD]; Voronoi tessellation of double cone domains **(D)** and their frequency distribution **(E)** [statistics (in 100 µm^2^) are the mean area (domain), its standard deviation (SD), the minimum (min) and maximum (max) areas, and the regularity index=mean/SD]. **(F–J)** Same presentation of data as per **(A–E)** but for the centroventronasal retina at 1170 ATU. **(K–O)** Same presentation of data as per **(A–E)** but for the centrodorsotemporal retina at 1170 ATU. **(P–S)** Same presentation of data as per **(B–E)** but for a random distribution of points matched for density with that in **(K)** and constrained by single cone soma size.”

now reads:

“Spatial analysis of single cone distributions from representative mosaics in the retina of Atlantic halibut undergoing metamorphosis. Single cones are marked with yellow dots. **(A–E)** Cone mosaic from the centrodorsonasal retina at 773 ATU **(A)**; Nearest neighbour analysis of double cone centroids, illustrating the near neighbours of a single cone, including its nearest neighbour in red **(B)** and their frequency distribution **(C)** [statistics (in µm) are the mean nearest neighbour distance, its standard deviation (SD), the minimum (min) and maximum (max) nearest neighbour distances, and the regularity index=mean/SD]; Voronoi tessellation of double cone domains **(D)** and their frequency distribution **(E)** [statistics (in 100 µm^2^) are the mean area (domain), its standard deviation (SD), the minimum (min) and maximum (max) areas, and the regularity index=mean/SD]. (**F**–**J**) Same presentation of data as per **(A–E**) but for the centroventronasal retina at 878 ATU. **(K–O)** Same presentation of data as per **(A–E)** but for the centrodorsotemporal retina at 878 ATU. **(P–S)** Same presentation of data as per **(B–E)** but for a random distribution of points matched for density with that in **(K)** and constrained by single cone soma size.”

The original Article has been corrected.

**Figure 1:**
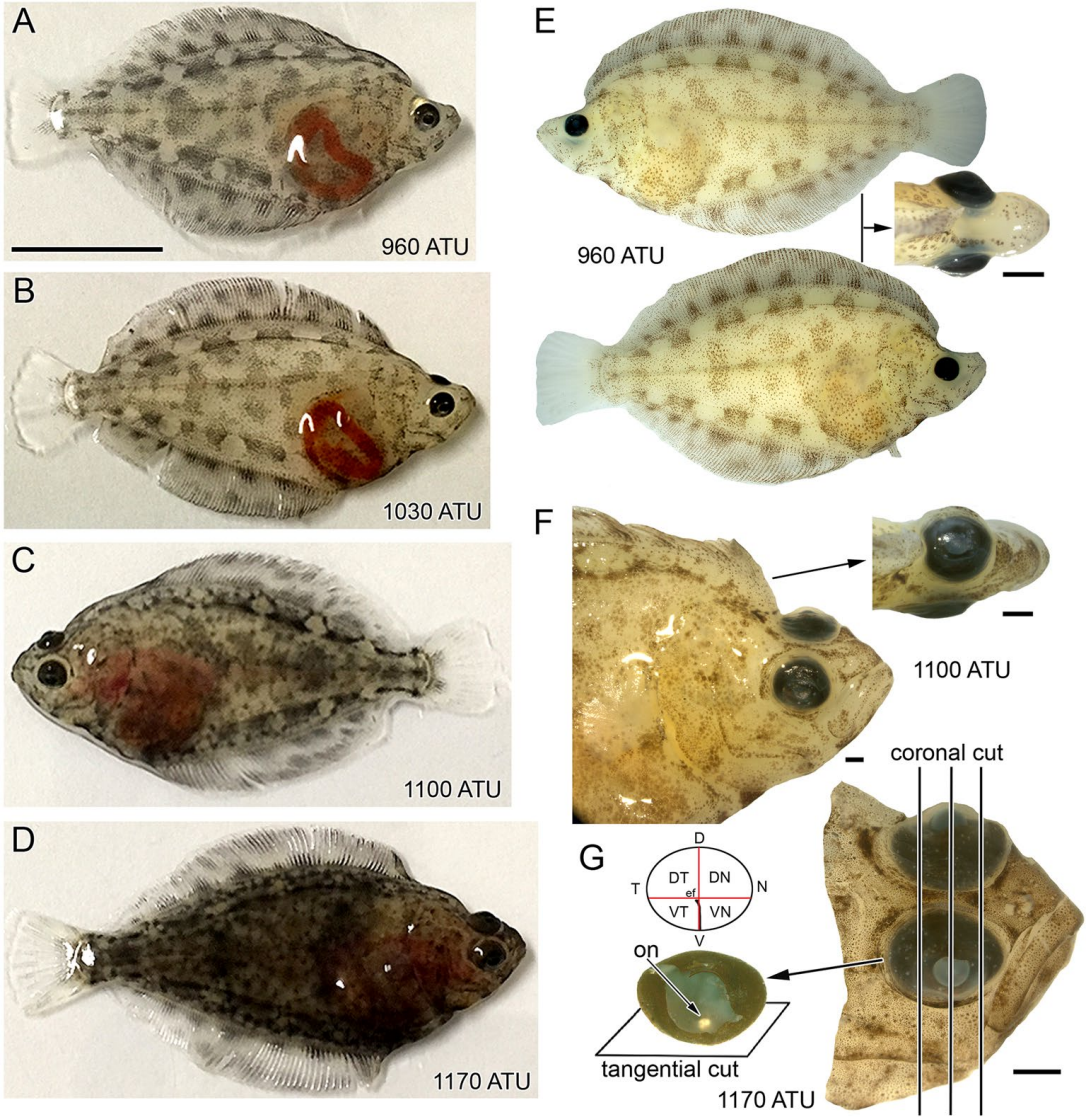
Developmental stages of Atlantic halibut examined illustrating eye migration and orientation of histological sectioning. (**A**–**D**) Body morphology of live fish at approximately 960 ATU (82 days post-hatching) (**A**), 1030 ATU (89 days post-hatching) (**B**), 1100 ATU (96 days post-hatching) (**C**), and 1170 ATU (103 days post-hatching) (**D**). The left eye is migrating in all fish except for the one shown in (**C**), where the right eye is migrating. (**E**) Lateral and top views of a fixed specimen at 960 ATU. Migration of the left eye has just started. (**F**) Lateral and top views of a fixed specimen at 1100 ATU. The left eye has migrated half way between the blind side (bottom part of the fish following metamorphosis) and ocular side (top part of the fish following metamorphosis). (**G**) Head of fixed specimen at 1170 ATU showing completed eye migration and the extracted right eye without lens. The optic nerve (on) exits the retina below the crossing of theoretical short and long ellipse axes that characterize this elliptically-shaped eye and retina. The optic nerve head coincides with the central end of the embryonic fissure (ef) which extends to the ventral periphery. The panel illustrates the direction of sectioning when full eyes were cut, revealing tangential sections of central retina and more oblique sections toward the periphery, and coronal cuts when a head was sectioned, revealing radial views of the retina throughout the lens region and tangential views at the nasal and temporal ends. The schematic shows the division of a right eye retina into quarters (VT, ventrotemporal; VN, ventronasal; DT, dorsotemporal; DN, dorsonasal). Other abbreviations: V, ventral; D, dorsal; N, nasal; T temporal. Scale bar in (A) equals 1 cm and holds for (**B**–**D**) and the lateral views in (**E**); the other scale bars represent 1 mm and are associated with the figures closest to them.

**Figure 2:**
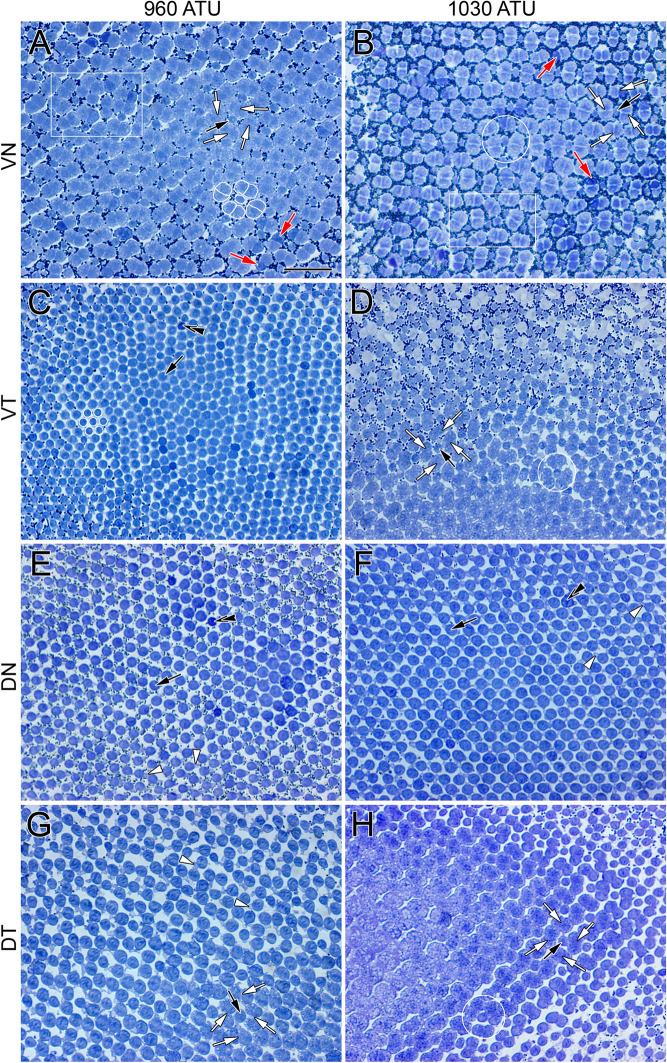
Micrographs of tangential EPON sections showing cone distributions at the level of largest ellipsoid cross section or near the base of the outer segments from various regions of the light adapted retina of Atlantic halibut undergoing metamorphosis. (**A**,**C**,**E**,**G**) Central area from the ventronasal (**A**), ventrotemporal (**C**), dorsonasal (**E**) and dorsotemporal (**G**) retina at 960 ATU. (**B**,**D**,**F**,**H**) Equivalent central sectors to those in (**A**,**C**,**E**,**G**) but from a retina at 1030 ATU. At 960 ATU, the ventronasal (**A**) and dorsotemporal (**D**) quadrants show cone square mosaics whereas honeycomb mosaics characterize the ventrotemporal (**C**) and dorsonasal (**E**) quadrants. (**A**) A square mosaic unit consisting of a single cone surrounded by four double cones is traced in white; white arrows point to the apposing partitions of double cone members surrounding a single cone (black arrow); red arrows indicate double cone members that stain darker than their counterparts. The area within the white rectangle illustrates a less regular arrangement of single and double cones. (**C**) A honeycomb mosaic unit consisting of one single cone surrounded by six others is shown in white; double black arrowheads point to a single cone that stains darker than most others in the field of view. (**E**,**G**) White arrowheads point to accessory outer segments of individual single cones. At 1030 ATU (**B**,**D**,**F**,**H**), only the central region of the dorsonasal quadrant retains a honeycomb mosaic (**F**). The white rectangle in (**B**) illustrates an area with less regular disposition of single and double cones. (**B**,**D**,**H**) White circles encompass a single cone flanked by a triangular arrangement of three neighbouring double cones. Abbreviations as per Fig. 1. Scale bar in (**A**) equals 10 µm and holds for all panels.

**Figure 6:**
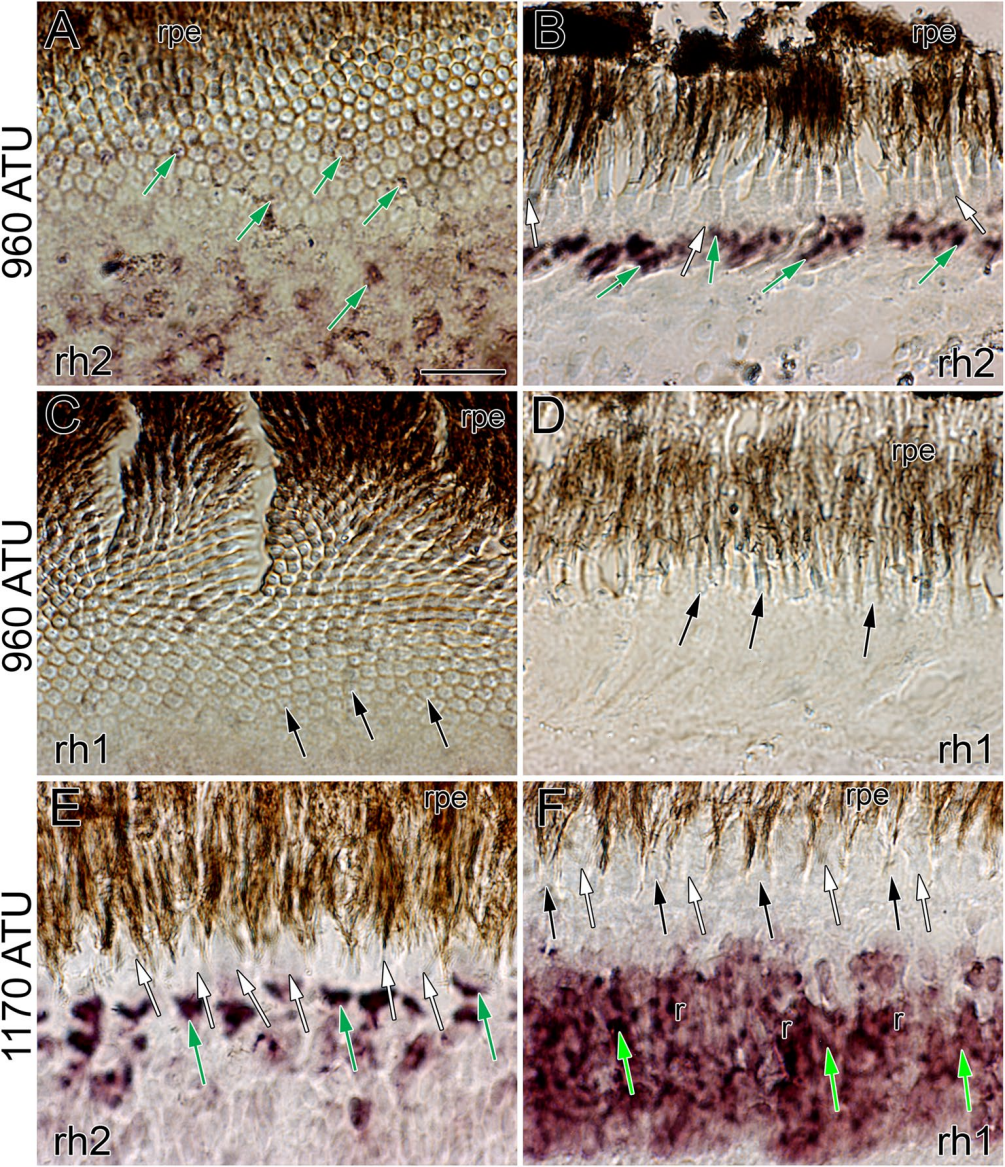
Micrographs of cryosections subjected to *in-situ* hybridization with the *rh1* and *rh2* riboprobes. (**A**) Tangential micrograph from the dorsonasal retina at 960 ATU showing labeling of *rh2* transcripts among single cones of the honeycomb mosaic (green arrows). (**B**) Radial micrograph from a square mosaic region of the centroventral retina showing prominent labeling of *rh2* transcripts (green arrows). (**C**,**D**) Tangential (**C**) and radial (**D**) micrographs from the dorsonasal retina at 960 ATU illustrating lack of *rh1* label among cones of the honeycomb mosaic. (**E**,**F**) Radial micrographs of the retina at 1170 ATUs showing *rh2* label of double cones (green arrows) (**E**) and *rh1* label of rods (r) (**F**). The *rh1* label is displaced vitreally (light green arrows) with respect to that of *rh2* in accordance with the position of rod myoids relative to those of cones in the light adapted retina. Other abbreviations and symbols as per Fig. 5. Scale bar in (**A**) equals 10 µm and holds for (**B**–**F**).

**Figure 7:**
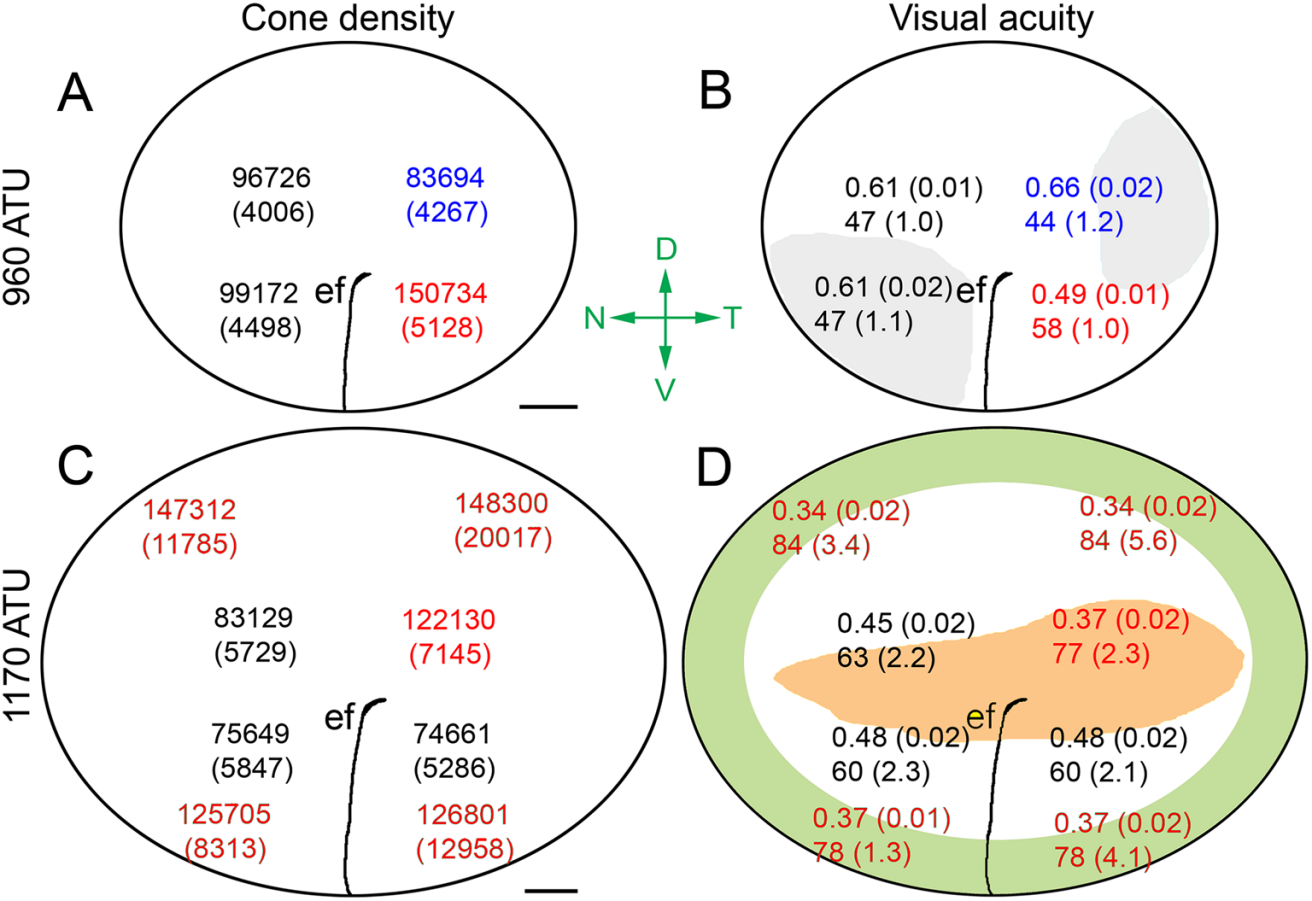
Diagrams illustrating topographic maps of cone densities and visual acuity-related variables extracted from the retinas of four Atlantic halibut at 960 ATU or 1170 ATU. Each statistic is the mean (± SD). (**A**,**C**) Cone density per mm^2^; gray zones in (**C**) delineate the approximate areas of square mosaic presence. (**B**,**D**) Minimum resolvable angle (º) (top number), and distance (in mm) (bottom number) at which a 0.5 mm target would be resolved. The green and pink zones in (**D**) denote the approximate areas associated with the peripheral square mosaic and triple cone presence, respectively. Means with different colour are statistically different at α = 0.05 level of significance. The embryonic fissure (ef) runs from the ventral retina toward the centro-temporal retina. Abbreviations (axes in green): D, dorsal; V, ventral; N, nasal; and T, temporal. Scale bars in (**A**) and (**C**) equal 0.2 mm.

