# Supplementary material for: Photoreceptor distributions, visual pigments and the opsin repertoire of Atlantic halibut (Hippoglossus hippoglossus)
Source: Sci Rep. 2022 May 16;12:8062. doi: 10.1038/s41598-022-11998-9 (PMC9110347; doi:10.1038/s41598-022-11998-9)
Supplement: Supplementary file 1 — Supplementary Information. [file 41598_2022_11998_MOESM1_ESM.pdf]

Supplementary Information

Photoreceptor distributions, visual pigments and the opsin repertoire of Atlantic halibut (*Hippoglossus hippoglossus*)

Kennedy Bolstad[1] & Iñigo Novales Flamarique[1,2*]

[1]Department of Biological Sciences, Simon Fraser University, Burnaby, British Columbia, V5A 1S6, Canada.

[2]Department of Biology, University of Victoria, Victoria, British Columbia, V8W 2Y2, Canada.

*Correspondence and requests for materials should be addressed to I.N.F. (email: inigo@sfu.ca) or K.B. (email: kbolstad@sfu.ca)

Supplementary Figures

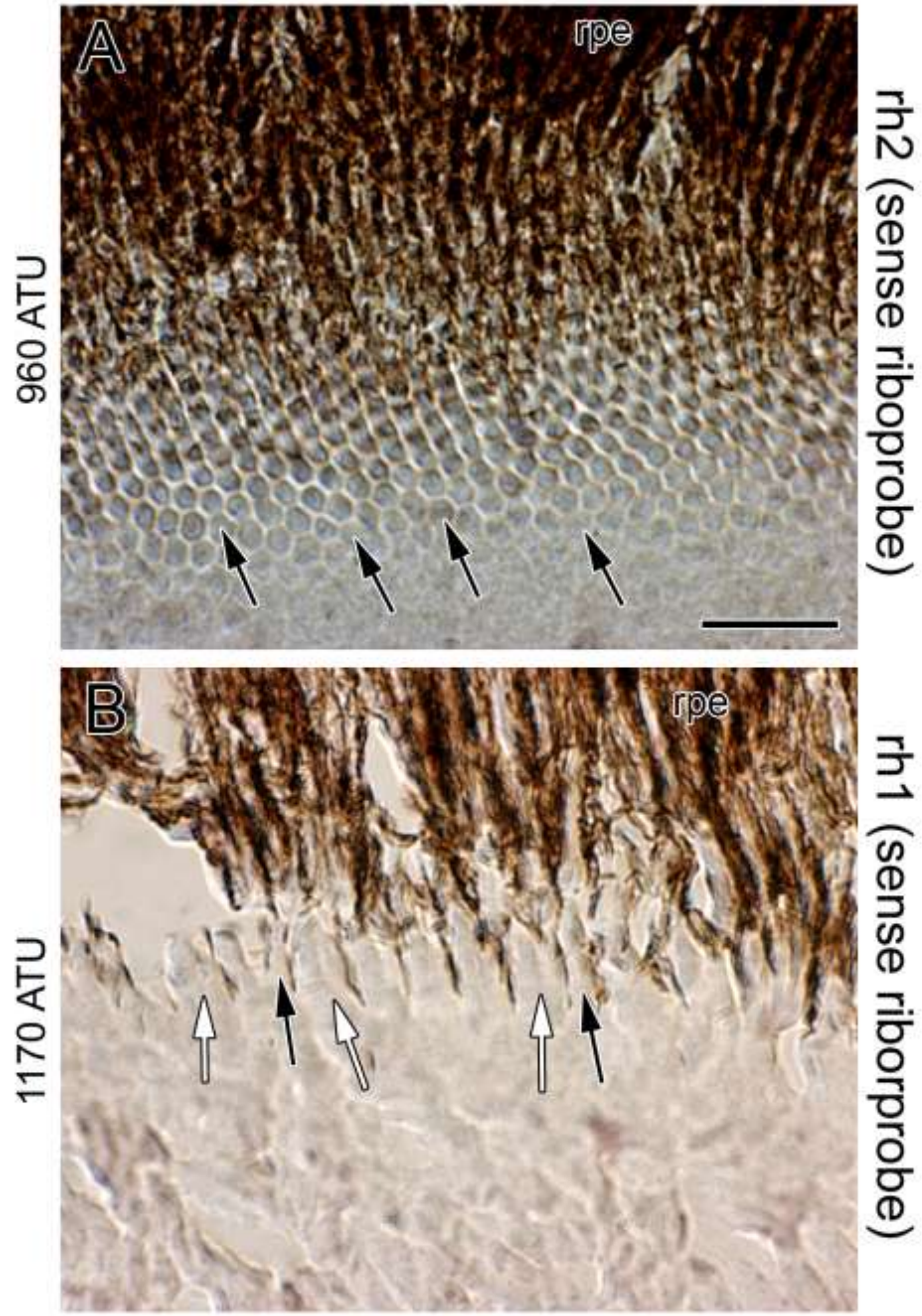

Supplementary Figure S1. Micrographs of Atlantic halibut following in-situ hybridization with *rh2* and *rh1*sense riboprobes. (A) Tangential section at 960 ATU exposed to *rh2* sense riboprobe. (B) Radial section at 1170 ATU stage exposed to *rh1* riboprobe. No labeling was apparent in either section. Black arrows point to single cones and white arrows point to the partitioning membranes between double cones. Abbreviation: rpe, retinal pigment epithelium. Magnification bar = 10µm.

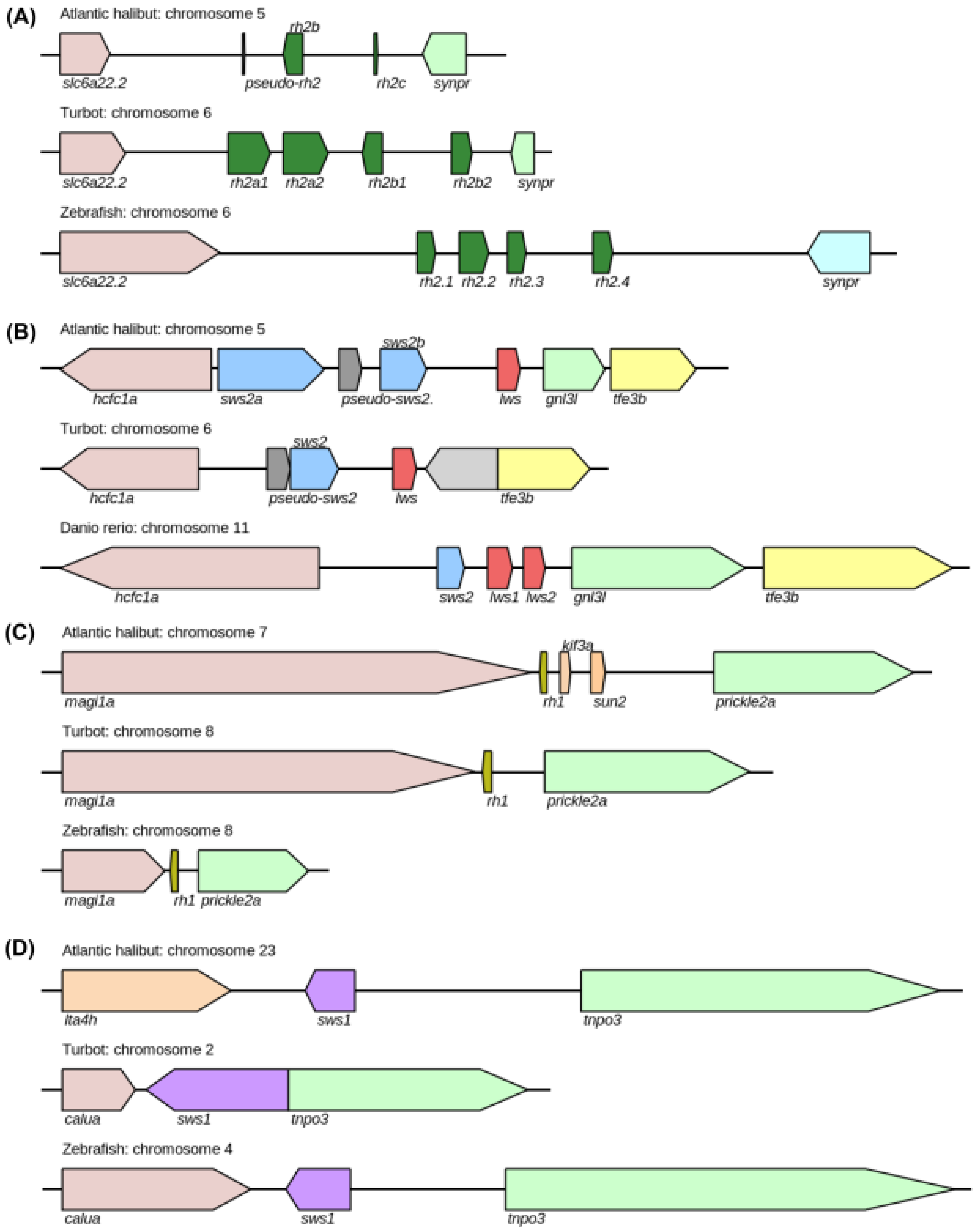

Supplementary Figure S2. Locations of predicted opsin coding sequences and their immediate flanking genes in Atlantic halibut (*Hippoglossus hippoglossus*) compared to turbot

(*Scophthalmus maximus*) and zebrafish (*Danio rerio*) for (A) *rh2* (B) *sws2* and *lws*, (C) *rh1*, and (D) *sws1*. Note that the *sws2-lws* gene cluster in turbot does not include *gnl3l* as the immediate flanking gene in the current NCBI annotation, but an uncharacterized protein (shown in grey). As such, for the *sws2-lws* gene cluster, two downstream flanking genes were shown.

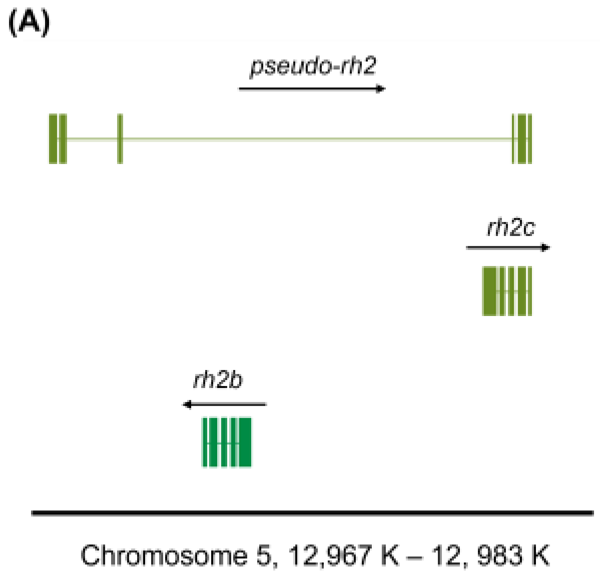

Supplementary Figure S3. Predicted *rh2* sequences in Atlantic halibut. (A) One of the predicted sequences (XP_034442261.1; *pseudo-rh2*) overlaps with the other two *rh2* sequences (accessions: XP_034442261.1 and XP_034442262.1) and has six exons, which is unusual for teleost *rh2* opsins. As such, we hypothesize that this sequence is a *pseudo-rh2* gene. (B) Refined *rh2* sequence structures, where only the first three exons of the *pseudo-rh2* sequence is retained. Opsin names included in this figure follow those that were assigned after phylogenetic analysis.

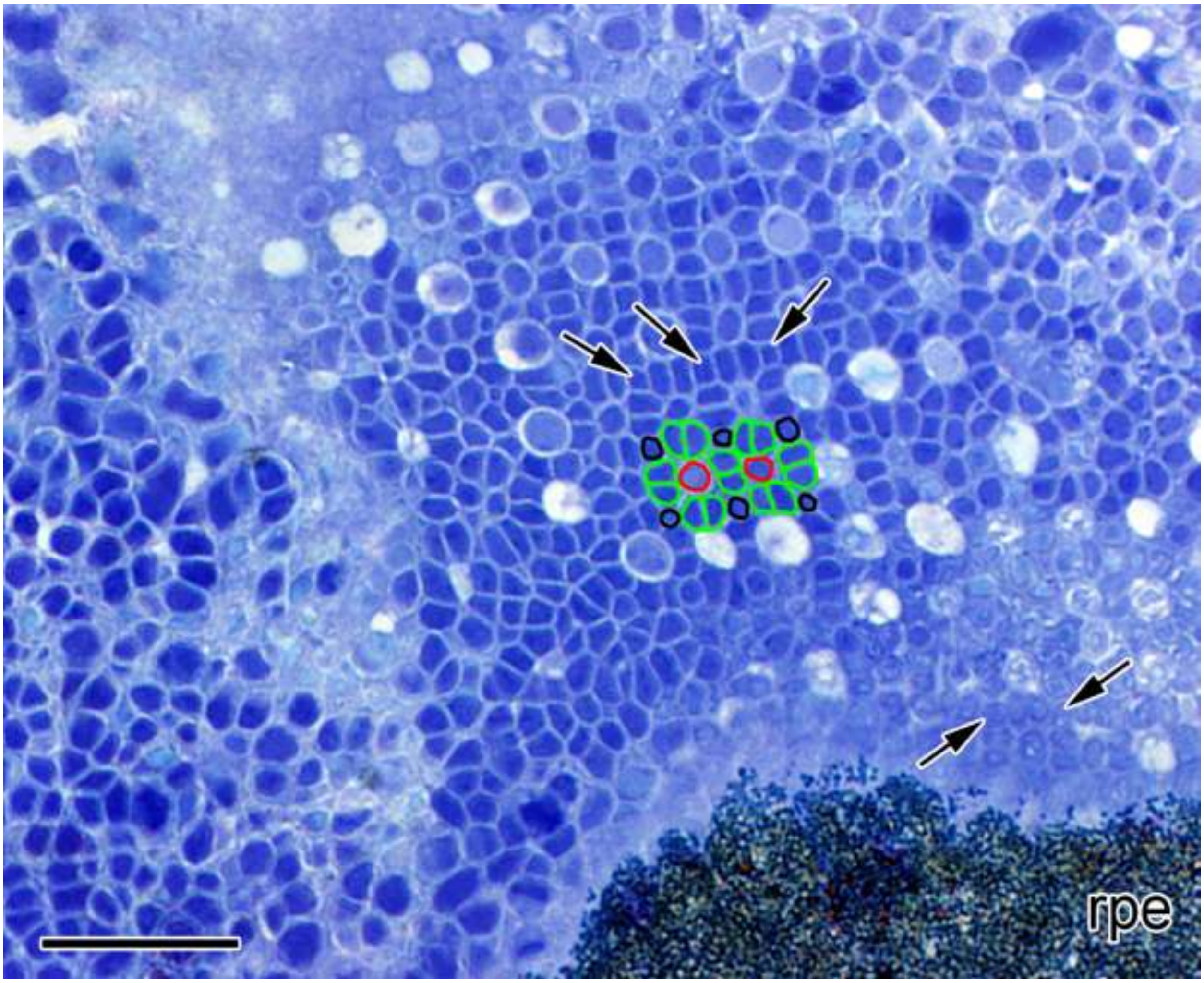

Supplementary Figure S4. Micrograph of Atlantic halibut retinal peripheral growth zone. The contours of two unit mosaics are traced showing double cones (green), single centre cones (red) and single corner cones (black). Black arrows point to other single corner cones. Abbreviation: rpe, retinal pigment epithelium. Magnification bar = 10µm.

Supplementary Tables

Supplementary Table S1. Predicted opsins from the Atlantic halibut reference genome[1] generated from BLASTp queries of zebrafish opsins (e-value < 1e-5, percent identity ≥50%). Included in the table are the opsin subclasses assigned after phylogenetic analysis.

**Opsin class**	**Opsin subclass**	**Protein accession**	**CDS chromosome: location**	**Gene ID**
SWS1	SWS1	XP_034434209.1	23 (complement): 18,346,408 to 18,348,321	*opn1sw1*
SWS2	SWS2A	XP_034440595.1	5: 23,451,385 to 23,452,968	*LOC117761093*
	SWS2B	XP_034441481.1	5: 23,458,711 to 23,462,900	*LOC117761574*
RH1	RH1	XP_034445498.1	7 (complement): 19,548,054 to 19,549,112	*LOC117764105*
RH2	RH2B	XP_034442261.1	5 (complement): 12,972,356 to 12,973,875	*LOC117761967*
	RH2C	XP_034442262.1	5: 12,981,421 to 12,982,946	*LOC117761968*
	pseudo- RH2	XP_034442263.1	5: 12,967,537 to 12,982,946	
LWS	LWS	XP_034441780.1	5: 23,469,900 to 23,471,991	*LOC117761719*

Supplementary Table S2. Predicted opsin proteins compared to published opsin proteins from Atlantic halibut[2]. Predicted opsin names follow those assigned after phylogenetic analysis.

**Published opsin**	**Predicted opsin**	**Opsin accession**	**No. of amino acids**	**Percent identity**	**Query cover (%)**	**E-value**
SWS1	SWS1	XP_034434209.1	339	100	100	0
SWS2	SWS2A	XP_034440595.1	353	99	100	0
	SWS2B	XP_034441481.1	353	75	100	0
RH1	RH1	XP_034445498.1	352	100	100	0
RH2	RH2B	XP_034442261.1	352	100	100	0
	RH2C	XP_034442262.1	352	86	100	0
LWS	LWS	XP_034441780.1	357	99	100	0

Supplementary Table S3. Amino acid sequence similarity between predicted and published[2] Atlantic halibut opsins. Predicted opsins with an identity ≥ 70% to published opsins are bolded. Predicted opsin names follow those assigned after phylogenetic analysis.

**Predicted opsins**	**Published Atlantic halibut opsins**				
	SWS1	SWS2	RH1	RH2	LWS
SWS1	**100**	46	47	48	45
SWS2A	47	**99**	49	54	42
SWS2B	46	**75**	50	52	41
RH1	46	50	**100**	62	38
RH2B	48	53	61	**100**	45
RH2C	50	53	61	**86**	45

LWS	45	42	39	45	**99**

Supplementary Table S4. Predicted opsin proteins from Atlantic halibut compared to corresponding visual opsins in zebrafish and nonvisual opsins from Atlantic halibut and zebrafish. Disulfide bridges are noted by C110 and C187, the chromophore binding site by K296, counterion residue at site 113, and motifs at sites 134-136[3]. See Supplementary Tables S11 and S13 for accession numbers. Predicted opsin names follow those assigned after phylogenetic analysis.

**Opsin class**	**Species**	**C110**	**E113 or Y113**	**ERW 134-136**	**C187**	**K296**
SWS1	Atlantic halibut SWS1	C	E	ERY	C	K
	Zebrafish	C	E	ERY	C	K
SWS2	Atlantic halibut SWS2A	C	E	ERW	C	K
	Atlantic halibut SWS2B	C	E	ERW	C	K
	Zebrafish	C	E	ERW	C	K
RH1	Atlantic halibut RH1	C	E	ERW	C	K
	Zebrafish RH1	C	E	ERW	C	K
RH2	Atlantic halibut RH2B	C	E	ERY	C	K
	Atlantic halibut RH2C	C	E	ERY	C	K
	Zebrafish RH2.1	C	E	ERY	C	K
	Zebrafish RH2.2	C	E	ERY	C	K
	Zebrafish RH2.3	C	E	ERY	C	K
	Zebrafish RH2.4	C	E	ERY	C	K
LWS	Atlantic halibut LWS	C	E	ERW	C	K
	Zebrafish LWS.1	C	E	ERW	C	K
	Zebrafish LWS.2	C	E	ERW	C	K

opn4m1/	Atlantic halibut	C	Y	DRY	C	K
melanopsin-1	Zebrafish	C	Y	DRY	C	K
opn4xb	Atlantic halibut	C	Y	DRY	C	K
opn4xb	Zebrafish	C	Y	DRY	C	K
tmt opn3a	Atlantic halibut	C	Y	ERY	C	K
	Zebrafish	C	Y	ERY	C	K

Supplementary Table S5. Key amino acid tuning sites for *sws1* opsins[4-6]. All amino acid sites reported are standardized to bovine rhodopsin.

Opsin name	Species	46	49	52	86	90	93	114	118
Predicted	Atlantic halibut SWS1	S	F	T	F	S	Q	S	S
SWS1	Japanese flounder	S	F	T	F	S	Q	S	S
	Spotted halibut	S	F	T	F	S	Q	S	S
	Barfin flounder	S	F	T	F	S	Q	S	S
	Marbled sole	C	F	T	F	S	Q	S	S
	Turbot	F	F	T	F	S	Q	A	S
	Zebrafish	F	I	T	F	S	Q	A	S

Supplementary Table S6. Key amino acid tuning sites for *sws2* opsins[4,5]. All amino acid sites reported are standardized to bovine rhodopsin.

Opsin Name	Species	46	52	91	93	94	116	269	292
Predicted	Atlantic halibut SWS2A	F	T	S	V	G	M	A	S
	Atlantic halibut SWS2B	F	T	S	T	C	T	A	S
SWS2A	Japanese flounder	F	T	S	V	G	M	A	A
	Spotted halibut	F	T	S	V	G	M	T	A
	Barfin flounder	F	T	S	V	G	M	T	A
	Marbled sole	F	T	S	V	G	M	A	A
SWS2B	Japanese flounder	F	T	S	T	C	T	A	S
	Spotted halibut	F	T	S	T	C	T	A	S
	Barfin flounder	F	T	S	T	C	T	A	S
	Marbled sole	F	S	S	T	C	T	A	S
SWS2	Common sole	I	S	T	V	G	M	A	A
	Senegalese sole	F	T	S	T	A	M	T	S
	Turbot	F	T	S	T	C	S	A	S
	Zebrafish	F	T	S	V	A	T	A	S

Supplementary Table S7. Key amino acid tuning sites for *rh1* opsins[4-7]. All amino acid sites reported are standardized to bovine rhodopsin. The following acronyms are used for each species: AH (Atlantic halibut), JF (Japanese flounder), SH (Spotted halibut), BF (Barfin flounder), MS (Marbled sole), TB (Turbot), CS (Common Sole), SS (Senegalese sole).

**Opsin name**	**Predicted**	**RH1**							
	**AH RH1**	**JF**	**SH**	**BF**	**MS**	**TB**	**CS**	**SS**	**ZB**
**83**	N	N	N	N	D	N	N	N	D
**90**	G	G	G	G	G	G	G	G	G
**96**	Y	Y	Y	Y	Y	Y	Y	Y	Y
**102**	Y	Y	Y	Y	Y	Y	Y	Y	Y
**113**	E	E	E	E	E	E	E	E	E
**118**	T	T	T	T	T	T	T	T	T
**122**	E	E	E	E	E	E	E	E	E
**124**	G	A	A	A	S	G	G	A	G
**132**	A	A	A	A	A	A	A	A	A
**164**	A	A	A	A	A	A	A	A	A
**183**	M	M	M	M	M	M	M	M	M
**194**	R	R	R	R	R	R	R	R	R
**195**	A	A	A	A	A	A	A	A	T
**207**	M	M	M	M	M	M	M	M	M
**208**	F	F	F	F	F	F	F	F	F
**211**	H	H	H	H	H	H	H	H	H
**214**	I	I	I	I	I	I	I	I	I
**253**	M	M	M	M	M	M	M	M	M
**261**	F	F	F	F	F	F	F	F	F
**265**	W	W	W	W	W	W	W	W	W
**269**	A	A	A	A	A	A	A	A	A

**289**	T	T	T	T	T	T	T	T	T
**292**	A	A	A	A	A	A	A	A	A
**295**	A	A	A	A	A	A	A	A	A
**299**	A	S	A	A	A	S	S	S	A
**300**	V	I	V	V	I	I	I	I	V

Supplementary Table S8. Key amino acid tuning sites for *rh2* opsins[4-6]. All amino acid sites reported are standardized to bovine rhodopsin.

Opsin name	Species	49	52	97	122	207	292
Predicted	Atlantic halibut RH2B	C	T	S	E	M	A
	Atlantic halibut RH2C	C	T	T	Q	L	A
RH2A1	Japanese flounder	C	F	S	Q	M	A
	Turbot	C	F	S	Q	L	A
RH2A2	Japanese flounder	C	F	T	Q	M	A
	Turbot	C	F	T	Q	M	A
RH2A	Marbled sole	C	T	S	Q	M	A
RH2B1	Turbot	C	T	S	E	M	A
RH2B2	Turbot	C	T	S	E	M	A
RH2B	Japanese flounder	C	T	S	E	M	A
	Spotted halibut	C	T	S	E	M	A
	Barfin flounder	C	T	S	E	M	A
	Marbled sole	C	T	S	E	M	A
RH2C	Japanese flounder	C	T	S	Q	L	A
	Spotted halibut	C	T	T	Q	L	A
	Barfin flounder	C	T	T	Q	L	A
	Marbled sole	C	T	T	Q	L	A
	Turbot	C	T	S	Q	L	A
RH2.1	Zebrafish	I	F	C	Q	M	A
RH2.2	Zebrafish	C	L	C	Q	M	A
RH2.3	Zebrafish	C	F	T	Q	M	A
	Common sole	C	L	T	Q	M	A
	Senegalese sole	S	L	T	Q	M	A
RH2.4	Zebrafish	C	F	T	E	M	A
	Common sole	C	T	S	E	M	A
	Senegalese sole	C	T	S	E	M	A

Supplementary Table S9. Key amino acid tuning sites for *lws* opsins[4-6]. All amino acid sites reported are standardized to bovine rhodopsin.

Opsin name	Species	164	181	261	269	292
Predicted	Atlantic halibut LWS	S	H	Y	T	A
LWS	Japanese flounder	A	H	Y	T	A
	Spotted halibut	A	H	Y	T	A
	Barfin flounder	A	H	Y	T	A
	Marbled sole	S	H	Y	T	A
	Turbot	P	H	Y	T	A
	Common sole	A	H	Y	T	A
	Senegalese sole	A	H	Y	T	A
LWS.1	Zebrafish	A	H	Y	T	A
LWS.2	Zebrafish	A	H	F	T	A

Supplementary Table S10. Predicted λmax for Atlantic halibut visual opsins based off of variable amino acid tuning sites. Residue number is standardized to bovine rhodopsin.

Opsin Name	Reference λmax (nm)	Amino acid substitutions at tuning sites (±nm shift in λ_{max})	Predicted λmax (nm)
SWS1	367 nm[8]	--	~367 nm
SWS2A	465.6 nm[9]	A292S (-8 nm)[10]	~457.6 nm
SWS2B	416 nm[8]	--	~ 416 nm
RH1	494 nm[8]	--	~494 nm
RH2B	506 nm[8]	--	~506 nm
RH2C	490 nm[8]	--	~490 nm
LWS	552 nm[8]	A164S (2-6 nm)[10]	~554-558 nm

Supplementary Table S11. NCBI and SoleaDB protein accession numbers used in this study. Previously predicted opsins[1] from Atlantic halibut (*Hippoglossus hippoglossus*) are also included.

Organism	Common name	Opsin name	Protein accession
Danio rerio	Zebrafish	SWS1	NP_571394.1
		SWS2	NP_571267.1
		RH1	NP_571159.1
		RH2.1	NP_571328.2
		RH2.2	NP_878311.1
		RH2.3	NP_878312.1
		RH2.4	NP_571329.1
		LWS.1	NP_001300644.1
		LWS.2	NP_001002443.1
Hippoglossus hippoglossus[1]	Atlantic halibut	SWS1	AAM17917.1
		SWS2	AAM17920.1
		RH1	AAM17918.1
		RH2	AAM17916.1
		LWS	AAM17921.1
Paralichthys olivaceus	Japanese flounder	SWS1	BAW35575.1
		SWS2A	BAW35586
		SWS2B	BAW35589
		RH1	BAW35583.1
		RH2A1	BAW79257.1
		RH2A2	BAW79258.1
		RH2B	BAW35578.1
		RH2C	BAW35580.1
		LWS	BAW35572.1
Pseudopleuronectes yokohamae	Marbled sole	SWS1	BCL84791.1
		SWS2A	BCL84789.1
		SWS2B	BCL84790.1
		RH1	BCL84792.1
		RH2A	BCL84786.1
		RH2B	BCL84787.1
		RH2C	BCL84788.1
		LWS	BCL84785.1
Scophthalmus maximus	Turbot	SWS1	AWO98359.1
		SWS2	AWP03347.1
		RH1	QDY91961

		RH2A1	QDY91963.1
		RH2A2	QDY91964.1
		RH2B1	QDY91965.1
		RH2B2	QDY91966.1
		RH2C	QDY91967.1
		LWS	AAQ02802.1
Solea senegalensis	Senegalese sole	SWS2	solea_v4.0_unigene481685
		RH1	solea_v4.1_unigene22024
		RH2.3	solea_v4.0_unigene28898
		RH2.4	solea_v4.0_unigene416478
		LWS	solea_v4.0_unigene34221
Solea solea	Common sole	SWS2	solea_solea_v1.1_unigene342597
		RH1	solea_solea_v1.1_unigene42150
		RH2.3	solea_solea_v1.0_unigene71547
		RH2.4	solea_solea_v1.0_unigene94070
		LWS	solea_solea_v1.1_unigene189057
Verasper variegatus	Spotted halibut	SWS1	BAW35573.1
		SWS2A	BAW35584
		SWS2B	BAW35587
		RH1	BAW35581.1
		RH2B	BAW35576.1
		RH2C	BAW35579.1
		LWS	BAW35570.1
Verasper moseri	Barfin flounder	SWS1	BAO93915.1
		SWS2A	BAO93916
		SWS2B	BAO93917
		RH1	BAO93912.1
		RH2B	BAO93913.1
		RH2C	BAO93914.1
		LWS	BAO93911.1

Supplementary Table S12. Opsins initially predicted by BLASTp queries of the Atlantic halibut reference genome[1] with zebrafish opsins. These predicted proteins were eliminated from further analyses after examination of their sequence structure and location. Note that the predicted opsin corresponding to RH1 (accession: XP_034447460.1) is actually an exo-rhodopsin protein but was initially classified as an RH1-like protein.

**Opsins class**	**Protein accession**	**Reason for elimination**
SWS1	XP_034434208.1	Two SWS1 proteins are predicted from the same region of the genome. Compared to zebrafish SWS1, this protein has a lower percent identity (62%) and query cover (81%) than the other protein (73% and 97%, respectively).
SWS2	XP_034440596.1	Two SWS2 proteins are predicted from the same genomic region. The proteins have comparable percent identities and query covers to Zebrafish SWS2, but the sequence corresponding to this protein has a 5' UTR region approximately 8.4 Mbp upstream of the first exon.
RH1	XP_034447460.1	Contains introns similar to exo-rhodopsin (*exorh)*, found in pineal glands of teleosts such as zebrafish[11] and Atlantic halibut[12].

Supplementary Table S13. Non- visual opsin protein accession numbers obtained from NCBI.

Nonvisual opsin name	Species	Protein accession
OPN4M1/ melanopsin-1	Atlantic halibut	AIG92840.1
	Zebrafish	ADN39430.1
OPN4XB	Atlantic halibut	XP_034451267.1
	Zebrafish	NP_001245152.1
Teleost multiple tissue opsin 3a (TMT OPN3A)	Atlantic halibut	XP_034453944.1
	Zebrafish	NP_001269303.1
VA opsin	Zebrafish	NP_571661.1

Supplementary References

1. Einfeldt, A. L., Kess, T., Messmer, A., Duffy, S., Wringe, B.F., Fisher, J. *et al.* Chromosome level reference of Atlantic halibut *Hippoglossus hippoglossus* provides insight into the evolution of sexual determination systems. *Mol. Ecol. Res.* **21**, 1686–1696 (2021).
2. Helvik, J. V., Drivenes, Ø., Næss, T. H., Fjose, A. & Seo, H. C. (2001b). Molecular cloning and characterization of five opsin genes from the marine flatfish Atlantic halibut (*Hippoglossus hippoglossus*). *Vis. Neurosci.* **18**, 767–780 (2001b).
3. Davies, W.I., Tamai, T.K., Zheng, L., Fu, J.K., Rihel, J., Foster, R.G., et al. An extended family of novel vertebrate photopigments is widely expressed and displays a diversity of function. *Genome Res.* **25,** 1666–1679 (2015).
4. Yokoyama, S. Evolution of dim-light and color vision pigments. *Annu. Rev. Genomics Hum. Genet.* **9**, 259–282 (2008).
5. Nakamura, Y., Mori, K., Saitoh, K., Oshima, K., Mekuchi, M., Sugaya, T., et al. Evolutionary changes of multiple visual pigment genes in the complete genome of Pacific bluefin tuna. *Proc. Nat. Acad. Sci. USA.* **110,** 11061–11066 (2013).
6. Wang, Y., Zhou, L., Wu, L., Song, C., Ma, X., Xu, S., et al. Evolutionary ecology of the visual opsin gene sequence and its expression in turbot (*Scophthalmus maximus*). *BMC Ecol. Evol.* **21,** 1–12 (2021).
7. Musilova, Z., Cortesi, F., Matschiner, M., Davies, W.I.L., Patel, J.S., Stieb, S.M., *et al*. Vision using multiple distinct rod opsins in deep-sea fishes. Science 364, 588–592 (2019).

8. Kasagi, S., Mizusawa, K., Murakami, N., Andoh, T., Furufuji, S., Kawamura, S., et al. Molecular and functional characterization of opsins in barfin flounder (*Verasper moseri*). *Gene* **556,** 182–191 (2015).

9. Kasagi, S., Mizusawa, K. & Takahashi, A. Green-shifting of SWS2A opsin sensitivity and loss of function of RH 2A opsin in flounders, genus *Verasper*. *Ecol. Evol.* **8**, 1399–1410 (2018).

10. Takahashi, Y. & Ebrey, T. G. Molecular basis of spectral tuning in the newt short wavelength sensitive visual pigment. *Biochemistry* **42**, 6025–6034 (2003).

11. Mano, H., Kojima, D. & Fukada, Y. Exo-rhodopsin: a novel rhodopsin expressed in the zebrafish pineal gland. *Mol. Brain Res.***73**, 110–118 (1999).

12. Eilertsen, M., Drivness, Ø., Edvardsen, R.B., Ebbesson, L.O.E., Helvik, J.V. Exorhodopsin and melanopsin systems in the pineal complex and brain at early developmental stages of Atlantic halibut (*Hippoglossus hippoglossus*). *J. Comp. Neurol.* **522**, 4003–4022 (2014).
